# Management of traumatic spinal cord injury in the Nordic countries: a multidisciplinary survey

**DOI:** 10.1186/s13049-025-01349-8

**Published:** 2025-03-24

**Authors:** Anders C. Feyling, Johan Undén, Niklas Marklund, Ilke Malak, Ramona Åstrand, Jussi P. Posti, Tor Brommeland

**Affiliations:** 1 Department of Anaesthesia and Intensive Care, Division of Emergencies & Critical Care, Oslo University Hospital Ullevål, Oslo, Norway; 2 Department of Operation and Intensive Care, Hallands Hospital Halmstad, Halmstad, Sweden; 3 Anesthesia and Intensive Care, Clinical Sciences, Lund University, Lund, Sweden; 4 Department of Clinical Sciences Lund, Neurosurgery, Lund University and Skåne University Hospital, Lund, Sweden; 5 Department of Orthopedic Surgery, Hallands Hospital Halmstad, Halmstad, Sweden; 6 Department of Neurosurgery, Copenhagen University Hospital, Rigshospitalet, Copenhagen, Denmark; 7 Neurocenter, Department of Neurosurgery and Turku Brain Injury Center, Turku University Hospital and University of Turku, Turku, Finland; 8 Neurosurgical department, Oslo University Hospital Ullevål, Oslo, Norway; 9 Institute of Clinical Medicine, University of Oslo, Oslo, Norway; 10 Department of Research & Development, Division of Emergencies & Critical Care, Oslo University Hospital, Oslo, Norway

**Keywords:** Survey, Spinal cord injury, Clinical management

## Abstract

**Background:**

Management of traumatic spinal cord injury is complex and depends on a multidisciplinary approach involving pre-hospital services, spinal surgery, intensive care unit treatment and specialized rehabilitation. International clinical practice guidelines for the handling of these patients offer specific recommendations regarding transportation, radiological investigations, timing of surgery, intensive care management and rehabilitation. We performed a comprehensive multicenter survey to assess the agreement between the Nordic countries on the different aspects of traumatic spinal cord injury management.

**Methods:**

Sequential, cross-sectional, structured survey comprising the key clinical domains (pre-hospital services, spinal surgery, intensive care management and rehabilitation) in all tertiary spine trauma centers in Sweden, Denmark, Norway, Iceland and Finland. Data are presented descriptively.

**Results:**

A total of 109 respondents from 22 Nordic centers were invited to take the survey, with a response rate of 90% (98/109). Overall, clinical practices were comparable within the domains. Prehospital services had similar practices for airway management, clinical spine clearance and patient transport. Preoperative magnetic resonance imaging was available to 33/35 of the spine surgeons (94%) on a 24/7 basis. This examination was considered mandatory prior to surgery by 66% (23/35) of the surgeons. Surgery was defined as early if performed within 24 h of the injury by all surveyed surgeons. Augmented blood pressure regimens were widely applied in the intensive care units, with mean arterial pressure targets varying between > 80 and > 90 mmHg. Postoperative thromboprophylaxis was administered within 48 h by all centers and rehabilitation policies were similar overall. Notable variations in practice were the occasional steroid administration and the use of lumbar drains in 54% (14/26) of intensive care units.

**Conclusion:**

Although there is some variability in the current management of traumatic spinal cord injury in the Nordic countries at the center- and country-level, practices in most key clinical domains are similar and follow established international guidelines.

**Supplementary Information:**

The online version contains supplementary material available at 10.1186/s13049-025-01349-8.

## Background

Traumatic spinal cord injuries (TSCI) are life-altering and potentially life-threatening injuries that often lead to significant and persistent disabilities in survivors [1]. These may include impaired motor and sensory functions, disruption of autonomic regulation with affected breathing, blood pressure control, bowel-, bladder-, and sexual functions that result in reduced social independence and quality of life [2]. Globally, an annual incidence of 10 to 83 TSCI cases per million has been reported, while European estimates vary between 10 and 37 per million population [3–6]. Recent studies indicate a change in the demographics of TSCI with fewer young patients involved in high-energy accidents, and more elderly patients with low-energy trauma mechanisms such as falls. [7–9].

The pathway from initial injury to the permanent, stabilized clinical outcome is influenced by acute and late management strategies [10]. Various international guidelines for patients with TSCI focus on pre-hospital care, timing of surgery, hemodynamic management and rehabilitation measures [11–16]. Although the Nordic countries are comparable in economic status, demographics and health care access, differences in medical management strategies may exist, and the area of TSCI has not been studied in this regard.

The Scandinavian Neurotrauma Committee (SNC) is an independent, not-for-profit organization comprised of neurosurgeons, neurointensivists, and neuroanesthesiologists from the Nordic countries dedicated to improving the care of patients with traumatic injuries to the central nervous system (CNS). The committee has previously published guidelines for the management of traumatic brain injuries [17, 18]. The aim of this survey was to evaluate current clinical practices for TSCI across centers in the Nordic countries. We hypothesized that there would be significant discrepancies in the practices due to the limited evidence base in the field of TSCI management.

## Methods

### Study design

This is a cross-sectional, observational survey study to assess the current clinical management of TSCI in all five Nordic countries. Initially, a preliminary survey was tested by six physicians from separate specialties. These practitioners were clinically active and had significant experience in the management of patients with TSCI within the Nordic region. Final adjustments to the questionnaire were made according to feedback from these physicians, and logistics of the survey were tested before digital distribution to respondents.

### Setting

All hospitals within the five Nordic countries with the capacity for tertiary management of TSCI were included in the study. To qualify, individual institutions were required to have access to the four domains of interest to the survey: (1) Pre-hospital care, (2) Intensive care unit (ICU) management, (3) Spine surgery (neurosurgery and/or orthopedic surgery), (4) TSCI rehabilitation. Respondents were all clinically active in the management of TCSIs and/or had a leading position within the clinic. The questionnaire was sent to one respondent per domain *and* hospital, with the exception of the surgical domain, which in some centers included both neurosurgeons and orthopedic surgeons.

### Data collection

The data was collected through a web-based survey written in esMakerNX3^®^ (Entergate AB) which was sent out to eligible respondents via e-mail with a unique identification token. The survey included a combination of single choice-, multiple choice-, and open-ended questions with the possibility of leaving comments after each section. No patient data was collected. The structure of the survey did not expose the respondents to identification. The entire questionnaire with responses can be found in Supplement 1.

### Statistical methods

Results are presented descriptively. Categorical data are reported as number of respondents for each category, with their proportions given as percentages. Questions with multiple choice answers of “Yes, always” and “Yes, sometimes” were considered equal responses and dichotomized as Yes while answers “Seldom” or “Never” were classified as No. This was done to identify clinically relevant disagreements between clinicians more clearly.

## Results

A total of 109 respondents were deemed eligible to take the survey and received an invitation. The response rate was 90% (98/109). Among these were 43 anesthesiologists (44%), 18 neurosurgeons (18%), 18 orthopedic surgeons (18%) and 15 physical medicine specialists or neurologists (15%). The remaining 4 physicians were specialists in emergency medicine or pediatrics. Participating centers are presented in Table 1. Fifty-five (59%) respondents reported working with a population coverage of 1 million or less. The key findings of the survey are summarized in Table 2.

**Table 1:** Participating institutions by country

Country (percentage of total responses)	Institutions
Denmark (13%)	Aarhus University Hospital Rigshospitalet
Finland (25%)	Helsinki University Hospital Kuopio University Hospital Oulu University Hospital Tampere University Hospital Turku University Hospital
Iceland (4%)	The National University Hospital of Iceland
Norway (19%)	Haukeland University Hospital Oslo University Hospital Ullevål St. Olav's University Hospital Stavanger University Hospital University Hospital of North Norway
Sweden (39%)	Karolinska University Hospital Linköping University Hospital Örebro University Hospital Ryhov County Hospital Sahlgrenska University Hospital Skåne University Hospital (Lund/Malmö) Södersjukhuset University Hospital of Umeå Uppsala University Hospital

**Table 2:** Key survey findings by country

	Question		DK	FIN	ICE	NOR	SWE	Total	%
**Pre-hospital**	Are paramedics allowed to intubate a patient with TSCI?	Yes No	0 4	0 5	0 1	0 4	0 7	0 21	0 100
	Does your service allow spinal clearance based on clinical assessment?	Yes No	2 2	5 0	1 0	1 2	7 0	16 4	80 20
	Is the primary referral institution a spinal trauma center?	Yes No	3 1	5 0	1 0	4 0	3 4	16 5	76 24
**ICU**	Are high-dose steroids routinely administered ?	Yes No	0 3	0 5	0 1	2 3	4 8	6 20	23 77
	Does your department routinely apply a MAP-protocol ?	Yes No	3 0	5 0	1 0	5 0	9 3	23 3	88 12
	What is the duration of targeted MAP-therapy?	1–3 days 3–7 days > 7 days	0 1 2	0 4 1	1 0 0	1 4 0	6 2 1	8 11 4	35 48 17
**Surgery**	Is MRI considered compulsory pre-op?	Yes No	4 0	9 1	1 0	7 0	12 1	33 2	94 6
	What is regarded as early surgery?	< 8 h < 24 h	2 2	3 7	1 0	3 4	6 7	15 20	43 57
	Is inititation of LWMH within 48 h?	Yes No	3 0	5 0	1 0	5 0	11 0	25 0	100 0
	Is lumbar drainage used for reduction in intraspinal pressure?	Yes No	0 4	1 9	0 1	1 6	2 11	4 31	11 89
**Rehab**	Is rehabilitation of patients with SCI undertaken in a specialized TSCI unit?	Yes No	2 0	3 1	0 1	3 0	6 0	14 2	87 13
	Is your unit accredited according to international standards like ISO or CARF	Yes No	1 1	0 3	0 1	2 1	4 2	15 1	47 53
	Is urodynamic examination available at your institution?	Yes No	2 0	4 0	1 0	3 0	6 0	16 0	100 0
	Does your department have access to botulinum injections and baclofen pump refilling/adjustments?	Yes No	2 0	4 0	1 0	3 0	6 0	16 0	100 0

Percentages are rounded to nearest integer for clarity

*DK* Denmark, *FIN* Finland, *ICE* Iceland, *NOR* Norway, *SWE* Sweden

### Pre-hospital care

A total of 21 respondents working in pre-hospital services replied to 13 questions with multiple choice answers. Not all questions were answered by all respondents. All advanced prehospital airway management was handled by physicians, with no sites reporting paramedics performing endotracheal intubation. Spinal clearance based on clinical assessment was applied by pre-hospital services in 16/20 (80%) of the sites. Clinical protocols used for spinal clearance were the NEXUS criteria and Canadian C-spine rules, reported by 11/15 (69%) and 2/15 (13%) of respondents, respectively. No single mode of spinal motion restriction was used exclusively in any country. Overall, the most commonly applied techniques were vacuum mattresses (17/21, 81%) and manual stabilization (12/18, 57%). Use of cervical collars was reported by 11/21 (52%) of respondents. Direct transfer from the accident scene to a tertiary center or the nearest center with spinal surgery service was preferred by 16/21 (76%) of the pre-hospital services.

### Intensive care unit management

A total of 26 physicians working in intensive care units answered 22 multiple choice questions. Not all questions were answered by all respondents. Protocols for augmenting mean arterial pressure (MAP) were reportedly used by 23 respondents (89%). Centers with such protocols reported MAP-targets of > 80 mmHg (55%), > 85 mmHg (32%) and > 90 mmHg (14%), respectively, with none targeting MAP > 95 mmHg. Duration of this therapy varied between centers, with 1–3 days (35%) and 4–7 days (48%) being the most common timeframes. Only four sites reported augmenting MAP beyond 7 days (17%). The vasoactive drug of choice was noradrenaline (22/25, 88%). Fluids routinely administered were predominantly crystalloids (16/26, 62%) followed by albumin (10/26, 39%). Maintaining hemoglobin thresholds for optimal spinal cord oxygenation was reported by 10/26 (39%). Routine use of high-dose steroids was not applied in any center. Occasional use of steroids was reported by two Norwegian and four Swedish centers. Physicians working in the ICU applied lumbar drainage “sometimes” for lowering intraspinal pressure in 14/26 (54%) of the respondents, but none did so routinely. Hyperthermia was considered detrimental to neurological outcome by 20/26 (77%) of respondents. The threshold for initiating treatment varied, but most centers (20/26, 95%) reported addressing temperatures exceeding 39 °C. Of the respondents, 8/25 (32%) initiated low-molecular weight heparin (LMWH) within 24 h and 17/25 (68%) between 24–48 h. Almost all sites reported starting enteral feeding within 48 h of surgery (25/26, 96%).

### Surgery

A total of 18 neurosurgeons and 18 orthopedic surgeons answered 36 multiple choice questions. Not all questions were answered by all respondents. Magnetic resonance imaging (MRI) was available 24/7 to 33/35 (94%) surgeons and was considered a mandatory radiological examination prior to surgery in all cases by 23/35 (66%) and “sometimes” by 10/35 (29%) of respondents. A standardized scheme for classification of spinal injury was applied by the majority of respondents, but 10/33 (30%) reported “never” using such tools. The most common classification systems were the Arbeitsgemeinschaft für Osteosynthesefragen (AO) and Thoracolumbar Injury Classification and Severity Score (TLICS) systems, reported by 65% and 22% respectively [19, 20]. Injuries at the cervical, thoracic and lumbar regions were operated on by orthopedic surgeons in 50%, 66% and 70% of cases, respectively. The use of skull traction for improving cervical alignment was evenly distributed across centers, with an overall use of 25/33 (76%). In all hospitals surgery was regarded as being performed early if done within the first 24 h by all hospitals. The neurological status of the patient was taken into account with regards to timing of surgery to at least some degree by 33/34 surgeons, with only one respondent answering “No, never”. Spinal navigation was used by 33/34 (91%) surgeons. Lumbar drainage of CSF was “always” or “sometimes” used by 4/35 (11%) of the respondents. Durotomy to lower intraspinal pressure was rarely done, with 34/35 (97%) never performing this procedure. The use of cervical collars post-operatively varied. Application in “all patients” was reported by 6/35 (17%) of surgeons, in “some patients” by 17/35 (49%) and “never” by 8/35 (23%) of the respondents. Initiation of LMWH post-operatively was permitted within 24 h by 17/34 (50%) of the surgeons, while 15/34 (44%) started within 24–48 h after surgery.

### Rehabilitation

A total of 16 physicians working in specialized rehabilitation centers replied to 20 multiple choice questions. Not all questions were answered by all respondents. Of the physicians, 5/16 (31%) were neurologists while the remaining were specialists in physical medicine. After the acute phase, long-term rehabilitation in a specialized unit was reported for “all” or “some” patients by 14/16 (88%) of respondents. Overall, 12/16 (75%) of the rehabilitation centers reported access to advanced physical devices such as weight-supported treadmills or exoskeletons for improved mobilization. This was evenly distributed across the countries. All units reported using botulinum toxin injections to treat urethral sphincter dysfunction as well as having the capability for Baclofen-pump adjustment and refilling for treatment of neurogenic spasms. The majority of rehabilitation centers reported having access to urodynamic examinations (94%), urotherapists (88%), and specialized personnel for bowel management (88%). The rehabilitation programs in use were accredited according to international standards (e.g., International Organization for Standardization or Commission on Accreditation of Rehabilitation Facilities) in 7/15 (47%) of the centers. Nearly all clinics (14/16, 88%) reported 1–3 months of thromboprophylaxis for patients with TSCI and severe neurological deficit (American Spinal Injury Association Impairment Scale (AIS) A, B, or C). LMWH was the main drug of choice (15/16, 94%). All respondents reported regular follow-ups for patients with TSCI. Overall, the duration of follow-up was life-long in 8/16 (50%) of centers.

## Discussion

This study surveyed the current practices of TSCI management in the five Nordic countries, encompassing pre-hospital services, intensive- and surgical care as well as rehabilitation. We present key findings with focus on topics identified as particularly important in international guidelines [12, 13, 15]. In most clinically relevant areas, broad similarities between countries and centers were observed, with some notable divergences in practice.

For the pre-hospital services, the majority of institutions allowed spinal clearance based on validated clinical tools such as the NEXUS and Canadian C-spine rules. Airway management with intubation was not performed by paramedics in any of the countries. Centers reported using varying techniques for spinal stabilization. Interestingly, nearly half of the pre-hospital services reported not routinely applying a cervical collar in patients with suspected TSCI. This is in accordance with current literature recommending different stabilization techniques and documenting a decline in the pre-hospital use of collars overall [21–23]. Direct transfer from the scene of accident to a hospital with spinal surgery capabilities in patients with isolated TSCI was the preferred choice for 76% of the respondents. This is also in line with guidelines recommending direct transport to a level I trauma center for those with suspected TSCI [24]. Transport to a local hospital first is associated with delayed surgery and less favorable neurological recovery [25, 26].

An elevated MAP may be beneficial for neurological outcome after TCSI [27]. While augmented blood pressure targets were widely applied in most centers in the intensive care phase, it is notable that three Swedish sites did not pursue this practice at all. Furthermore, the exact MAP-targets and duration of intervention varied somewhat between sites and countries. These disparities in practice are not clearly understood, but could partly be due to the limited evidence for hemodynamic therapy, as well as the increased intensive care burden associated with this treatment [28]. A recent guideline now calls for a MAP-targeted range of 75–80 mmHg as a lower limit and 90–95 mmHg as an upper limit, for 3–7 days [13].

Steroid treatment is considered ineffective following TSCI and is associated with increased risk of adverse events [29]. Despite this, 23% of intensivists and 17% of surgeons in this study reported “sometimes” administering high-dose steroids for neuroprotection. It is possible that individual clinicians still give some credence in early randomized controlled trials (RCTs) suggesting a possible benefit of steroids [30]. Apart from these differences, the overall administration of steroids was infrequent and limited to occasional use.

Timing of surgery for TSCI is debated. Current practice guidelines and recent reviews recommend surgery within 24 h of injury [12, 14, 15]. In our study, surgery within 8 h of injury was regarded as early by almost half of the respondents, with the remaining centers reporting the 24-h time frame. This suggests that the concept of early surgery is widely accepted in the Nordic countries. However, reports demonstrate that this is difficult to carry out in practice for logistical reasons [25, 31].

The use of LMWH to reduce the risk of venous thrombosis in patients with TSCI is recommended [32, 33]. The timing of LMWH initiation may have to be individually tailored as some patients with TSCI also have other injuries, such as intracranial hematoma at risk of expansion. In patients with isolated TSCI, early start of LMWH is safe [34]. This practice seems to have been adopted by clinicians in the Nordic countries, as 32% of intensivists and 50% of the surgeons reported starting LMWH within 24 h, and the remaining within 24–48 h.

Early rehabilitation of patients with TSCI is an important part of treatment [35, 36]. Current strategies promote early stabilizing surgery allowing mobilization already in the ICU. Direct transfer of patients with TSCI to a specialized unit for spinal cord injury (“unbroken chain”) seems to improve the final neurological result [37]. Such units exist in all Nordic countries except Iceland, due to its small population size. Even though a direct transfer between a level I trauma center and a specialized rehabilitation center is optimal, capacity of these units is limited and some patients are transferred to local hospitals first (“broken chain”) [36–38].

This study has some important limitations. It cannot be guaranteed that all respondents´ answers adequately reflected practices at their own site. Due to the relatively low numbers of participating centers, there is a risk of reporting bias that can only be addressed by significantly expanding the number of respondents for each site and domain. This would be logistically challenging, especially among relatively small nations with few eligible centers. The small overall sample limits the external validity of the findings. Not all questions were answered by all respondents in this survey. However, the response frequency remained high for almost all questions. The lower-than-expected response rate for a limited number of queries probably has varying explanations. However, we do not expect the overall conclusions to have been significantly affected by these missing answers. In the interest of brevity and focus, we chose our questions carefully by group consensus. Our final selection aimed to cover known key areas of interest, as well as subjects where we expected significant divergence or convergence in practice. As a results of these judgments, we recognize that some topics of potential clinical relevance were not addressed specifically. For instance, the subjects of antibiotic therapy and -prophylaxis, as well as ventilatory strategies in the ICU, were not covered in this survey.

## Conclusions

Some differences in management of TSCI among the Nordic countries and centers were observed. However, we found the overall agreement within most clinical domains to be considerable and practices generally compliant with internationally recognized guidelines. Our hypothesis of clinically relevant divergence among individual centers and Nordic countries was not observed.

## Supplementary Information

Supplementary Material 1.

## Data Availability

The entire survey with answers is available as Supplement 1

## Abbreviations

AIS: American spinal injury association impairment scale
AO: Arbeitsgemeinschaft für Osteosynthesefragen (Association of the Study of Internal Fixation)
ICU: Intensive care unit
MAP: Mean arterial pressure
NEXUS: National emergency X-radiography utilization study
SNC: Scandinavian neurotrauma committee
TLICS: Thoraco-lumbar injury classification
TSCI: Traumatic spinal cord injury
