# Supplementary material for: Management of traumatic spinal cord injury in the Nordic countries: a multidisciplinary survey
Source: Scand J Trauma Resusc Emerg Med. 2025 Mar 24;33:51. doi: 10.1186/s13049-025-01349-8 (PMC11934776; doi:10.1186/s13049-025-01349-8)

# Traumatic Spinal Cord Injury_FIN_PUB

## 1. In which country do you work?

| Namn | Antal | % |
|---|---|---|
| Denmark | 13 | 13,3 |
| Finland | 24 | 24,5 |
| Iceland | 4 | 4,1 |
| Norway | 19 | 19,4 |
| Sweden | 38 | 38,8 |
| **Total** | **98** | **100** |

| Svarsfrekvens |
|---|
| 100% (98/98) |

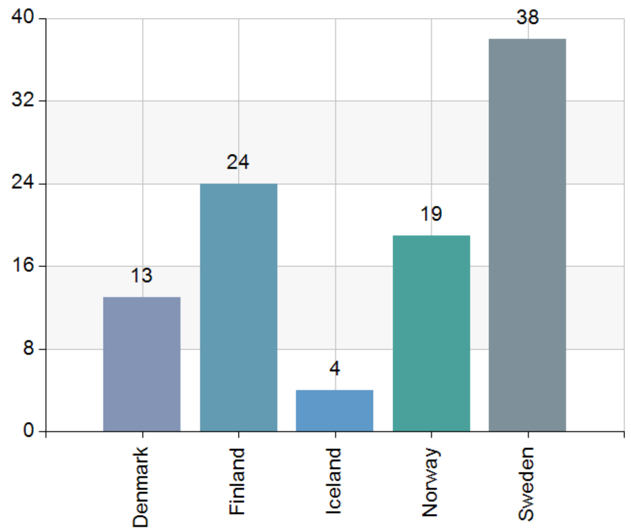


## 2. What is your medical specialty?

| Namn | Antal | % |
|---|---|---|
| Neurosurgery | 18 | 18,4 |
| Orthopedic surgery | 18 | 18,4 |
| Anaestesiology | 43 | 43,9 |
| Neurology | 5 | 5,1 |
| Physical rehabilitation | 10 | 10,2 |
| Other - Please specify | 4 | 4,1 |
| **Total** | **98** | **100** |

| Svarsfrekvens |
|---|
| 100% (98/98) |

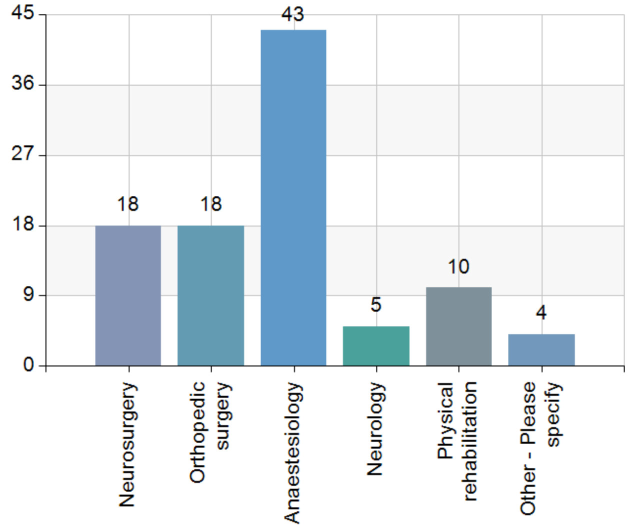


| **Other - Please specify** |
|---|
| Emergency medicine |
| Anesthesia and prehospital care |
| pediatric |
| Emergency Medicine |

## 3. I work in the following field of medicine:

| Namn | Antal | % |
|---|---|---|
| Pre-hospital | 21 | 21,4 |
| Intensive care unit | 26 | 26,5 |
| Neurosurgery | 15 | 15,3 |
| Orthopedic surgery | 20 | 20,4 |
| Rehabilitation | 16 | 16,3 |
| **Total** | **98** | **100** |

| Svarsfrekvens |
|---|
| 100% (98/98) |

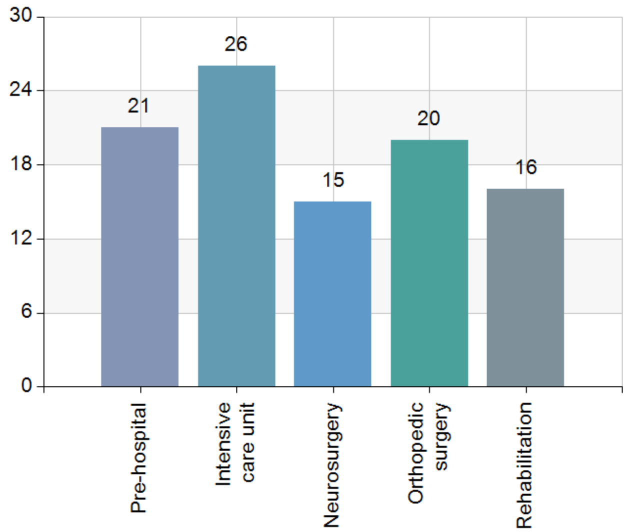


4. Approximately, what size of population does you hospital cover for patients with SCI (Spinal Cord Injury)?

| Namn | Antal | % |
|---|---|---|
| < 500,000 | 20 | 21,5 |
| 500,000 - 1 mill | 35 | 37,6 |
| 1 - 3 mill | 37 | 39,8 |
| 3 - 5 mill | 1 | 1,1 |
| >5 mill | 0 | 0 |
| **Total** | **93** | **100** |

| Svarsfrekvens |
|---|
| 94,9% (93/98) |

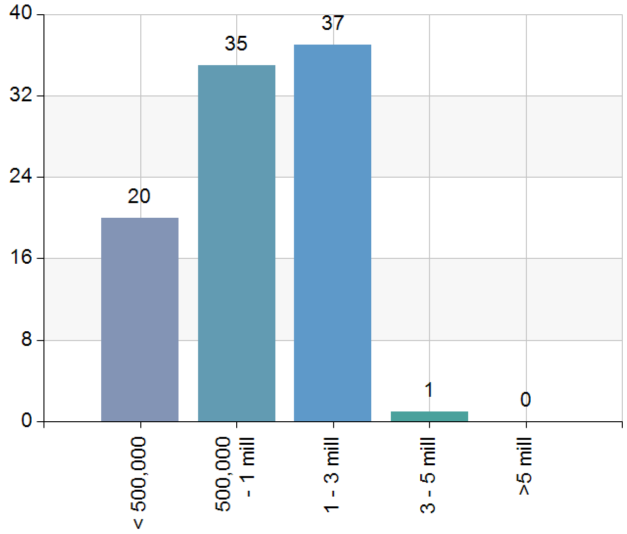


5. Approximately, how many cases of SCI (including central cord syndrome) does your department handle per year?

| Namn | Antal | % |
|---|---|---|
| <10 | 18 | 20,2 |
| 10-20 | 33 | 37,1 |
| 20-30 | 19 | 21,3 |
| 40-50 | 10 | 11,2 |
| 50-60 | 1 | 1,1 |
| >60 | 8 | 9 |
| **Total** | **89** | **100** |

| Svarsfrekvens |
|---|
| 90,8% (89/98) |

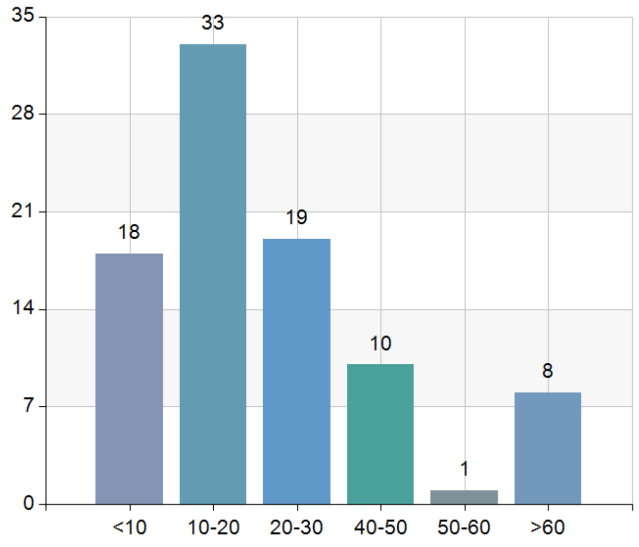


6. At your hospital, which medical specialty performs the surgery in patients with SCI at the following anatomical regions?

- Cervical

| Namn | Antal | % |
|---|---|---|
| Neurosurgeon | 43 | 50 |
| Orthopedic surgeon | 20 | 23,3 |
| Both Neurosurgeon and Orthopedic surgeon. | 23 | 26,7 |
| **Total** | **86** | **100** |

| Svarsfrekvens |
|---|
| 87,8% (86/98) |

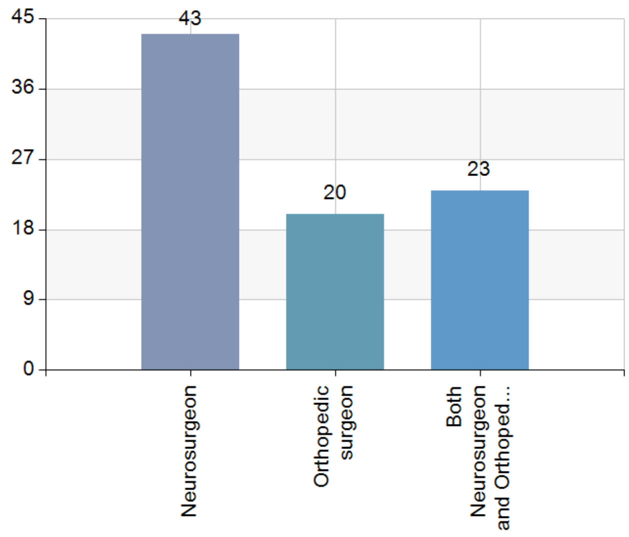


- Thoracic

| Namn | Antal | % |
| --- | --- | --- |
| Neurosurgeon | 6 | 7,1 |
| Orthopedic surgeon | 55 | 65,5 |
| Both Neurosurgeon and Orthopedic surgeon. | 23 | 27,4 |
| **Total** | **84** | **100** |

| Svarsfrekvens |
| --- |
| 85,7% (84/98) |

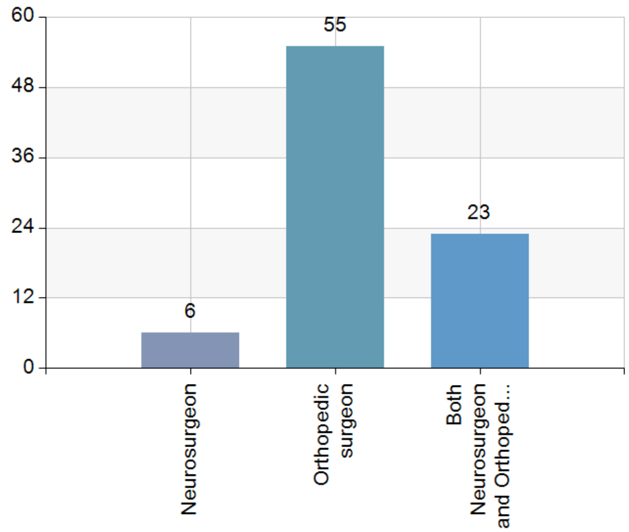


- Lumbar

| Namn | Antal | % |
| --- | --- | --- |
| Neurosurgeon | 5 | 5,8 |
| Orthopedic surgeon | 60 | 69,8 |
| Both Neurosurgeon and Orthopedic surgeon. | 21 | 24,4 |
| **Total** | **86** | **100** |

| Svarsfrekvens |
| --- |
| 87,8% (86/98) |

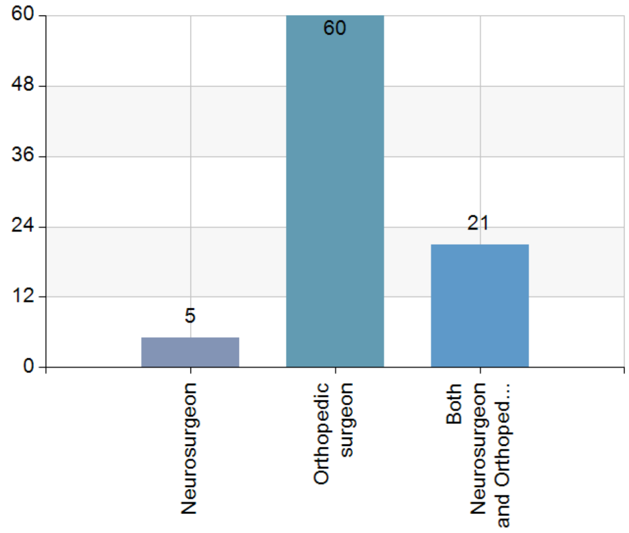


7. Are patients transferred directly to a specialized center for SCI rehabilitation after completing treatment at your hospital?

| Namn | Antal | % |
|---|---|---|
| Yes, always | 39 | 44,8 |
| Yes, sometimes | 34 | 39,1 |
| No - Transferred to local hospitals | 2 | 2,3 |
| Other - Please specify | 12 | 13,8 |
| **Total** | **87** | **100** |

| Svarsfrekvens |
|---|
| 88,8% (87/98) |

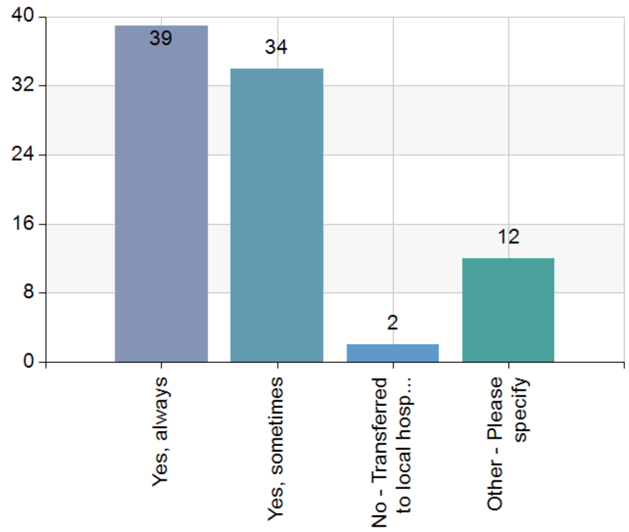


| Other - Please specify |
|---|
| I work prehospitally in HEMS. All our patients are directly flown to our traumacenter (definitive care). |
| Yes, most of the times |
| Our patients are not transferred primarily to our hospital for stabilisation |
| We are a specialized center from SCI rehab, so no transfer, but patients usually go directly to our department from neurosurgery or orthopedics. |
| I'm not sure of this, as I work in pre-hospital only |
| Directly to local hospital but later to SCI rehab in our hospital |
| "Yes, almost always" better reflects our situation. |
| I dont know |
| Our hospital (Tampere University Hospital) is one of the three university hospitals in Finland specialized in SCI care and rehabilitation |
| Within the hospital is a specialized center for SCI rehab, hence they are not transferred to a different hospital |
| ll patients except very old (>80-85 yrs) and very young (<5-7 yrs) COME diretly to us (specialized SCI Rehab center, at Grensás). We take care of Rehab of all "other" SCI patients in Iceland. (i.e., "Yes always, but the pat.s are referred to us). |
| All SCI is cared for at Landspitalinn (only centre with neurosurgery). Primary rehabilitation is at one of two main rehabilitation centres in Iceland. Both are capable of caring for SCI patients. |

• Do you have any comments related to the questions?

| |
|---|
| Patients are always assessed by a specialized center for SCI rehabilitation, but sometimes rehabilitated at their own hospital (at a rehabilitation clinic not specialized in SCI) |
| Goal/ intention is every patient, but there are cases of indirect transfers. |
| SCI patients are operated by the spine unit at the orthopedic dept. This is however a combined unit comprising both neuro and orthopaedic surgeons |
| You should have a alternative for other on the questions covering the operation specialities. Most hospitals in Sweden doesn't have thoracic and neurosurgeons. |
| Answers for the prehospital care in Region Stockholm. Q 5,6,7 not applicable |

| |
|---|
| The specialized center is contacted directly for discussion when the patient can be transferred from our hospital to the center. |
| Question number 5 is slightly unspecific, but I assume you mean newly injured patients. Apart from those we have about 600 SCI patients that we follow up life-long and take in if need for secondary rehab arises. |
| Things are about to change due to national regulations. There will be a specialized center for SCI rehabiliation soon |
| If directly means no other stop on the way then yes. If directly means promptly then no, there is often a waiting periode before the sci rehab center can accept the patient, most often 3-6 weeks. |
| No |
| As ambulance service we cover the region of Skåne - not directly conected to any hospital |
| The neurosurgeons are not involved in the surgery of the traumatic SCI. They might be consulted if there is an injury on the dura. |
| 2018-2021 så får jag följande:<br>Nyskadade patienter totalt 172, genomsnitt 43/år.<br>Nyskadade traumatiskt skadade patienter 107, genomsnitt 27/år.<br>Nyskadade icke-traumatiskt skadade patienter 65, genomsnitt 16/år. |
| SCI patients are admitted to neuro-ICU och evaluated/planned by neurosurgeon and ortohopedic spine surgeon for the surgical procedure, most cases are co-operated but some are operated by neurosurgeons or ortopedic spine surgeons alone. |
| Question 6, operations: Almost always (with few exceptions), a neurosurgeon AND an orthopedic surgeon operate together, as mentioned in the answer, but of course, the degree of input from each specialist varies with level of injury, as well as on an individual case-to-case basis. In cervical SCI, the neurosurgeon does more, while in lumbar (and thoracic) surgery, where the op. is mainly stabilization with appropriate osteosynthesis material, the orthopedic surgeon does more, sometimes much more, and the neurosurgeon is more ”assisting”. But close cooperation is the key word to what we here in Iceland think is a very successful approach.<br><br>Regarding question 7: See comment: alternative 1 is prehaps the most appropriate answer, but we are THE RECEIVERS (the most specialized center for SCI rehabilitation in Iceland). All patients (except the very young, the vey old, and occasional mild or very severe or complex multitrauma cases, dropouts due to lack of compliance (e.g., due to drug or alcohol addiction) and other exceptional odd cases) are transferred directly to us, as soon as they have became medically stable enough not to need to be in the acute hospital any more. |
| Somethimes a collaberation Orto spine surgeon an neurosurgeon |
| In Töölö hospital patients with central cord syndrome are usually operated by neurosurgeons but all traumatic SCIs are treated by orthopaedic surgeons. |
| Neurosurgeons are involved if neurologic symptoms are present. |
| ikke så relevante spørgsmål for præhospitalt miljø |
| As a prehospital provider we only treat at transport patientes |

- Are paramedics allowed to intubate a patient with SCI (Spinal Cord Injury) if airways need to be secured?

| Namn | Antal | % |
|---|---|---|
| Yes | 0 | 0 |
| No | 21 | 100 |
| **Total** | **21** | **100** |

| Svarsfrekvens |
|---|
| 100% (21/21) |

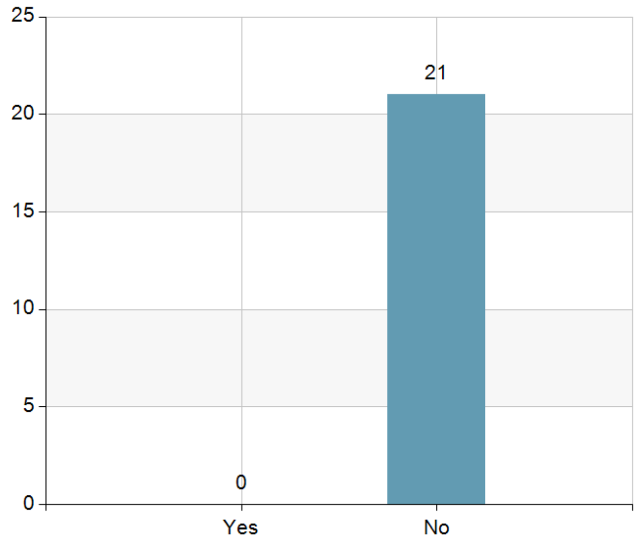


• What method(s) are most commonly used for stabilizing the cervical spine?

| Namn | Antal | % |
|---|---|---|
| Cervical collar | 11 | 52,4 |
| Vaccuum matress | 17 | 81 |
| Manual stabilization | 12 | 57,1 |
| Head blocks | 9 | 42,9 |
| Tape | 4 | 19 |
| Other - Please specify | 2 | 9,5 |
| **Total** | **55** | **261,9** |

| Svarsfrekvens |
|---|
| 100% (21/21) |

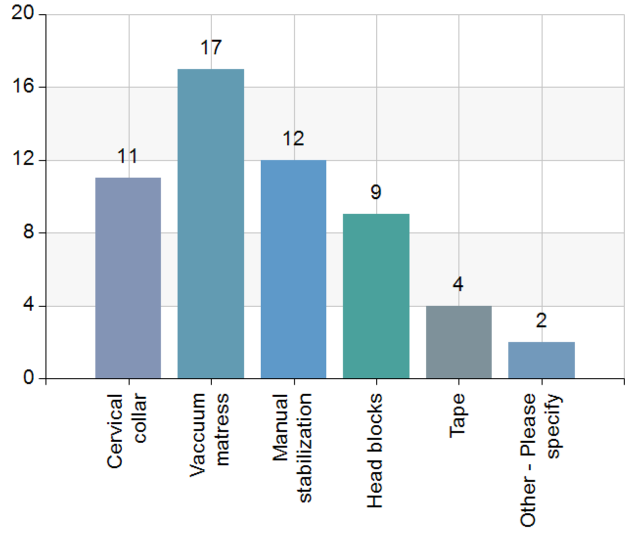


| **Other - Please specify** |
|---|
| Time critical patients = minimal spinal motion restriction. No backboard, vaccuum pillow on stretcher. Non-time critical patients = full motion restriction on scoop stretcher, no cervical collar. |
| All above methods are used but no specific method is ”mandatory”. Icelandic guidelines for prehospital treatment of SCI are modelled on the Norwegian guidelines. They stipulate careful handling and if indicated ”protection” (rather than ”stabilisation”) of the cervical spine (and rest of spine) by the most appropriate method according to patient and clinical circumstances. C-collar may be used but is not ”mandatory”. |

• Are backboards routinely applied in patients with SCI?

| Namn | Antal | % |
|---|---|---|
| Most often | 6 | 28,6 |
| Sometimes | 3 | 14,3 |
| Seldom | 8 | 38,1 |
| Never | 4 | 19 |
| **Total** | **21** | **100** |

| Svarsfrekvens |
|---|
| 100% (21/21) |

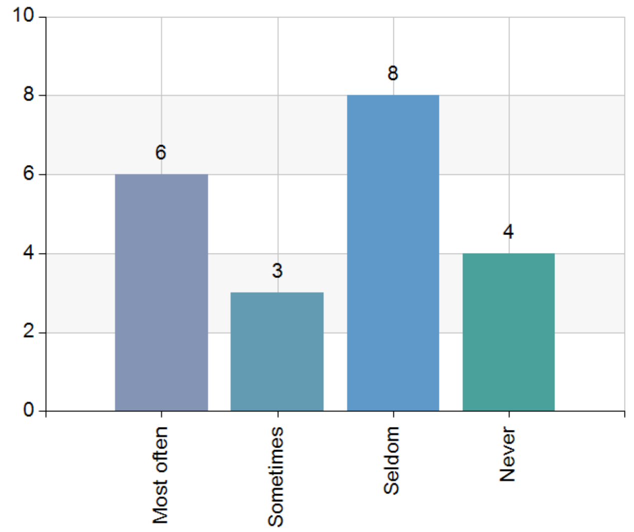


- Does your service allow spinal clearance based on clinical assessment?

| Namn | Antal | % |
|---|---|---|
| Yes | 16 | 80 |
| No | 4 | 20 |
| **Total** | **20** | **100** |

| Svarsfrekvens |
|---|
| 95,2% (20/21) |

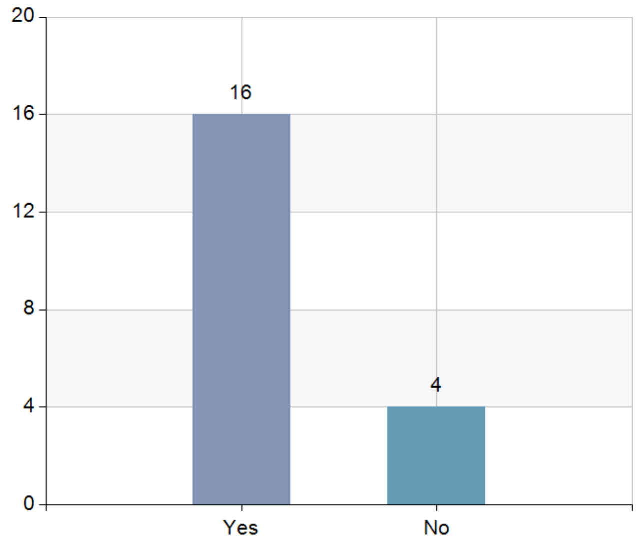


- If so, what clinical tool is most often used? [text]

| Namn | Antal | % |
|---|---|---|
| NEXUS | 11 | 68,8 |
| Canadian C-Spine | 2 | 12,5 |
| Other - Please specify | 3 | 18,8 |
| **Total** | **16** | **100** |

| Svarsfrekvens |
|---|
| 76,2% (16/21) |

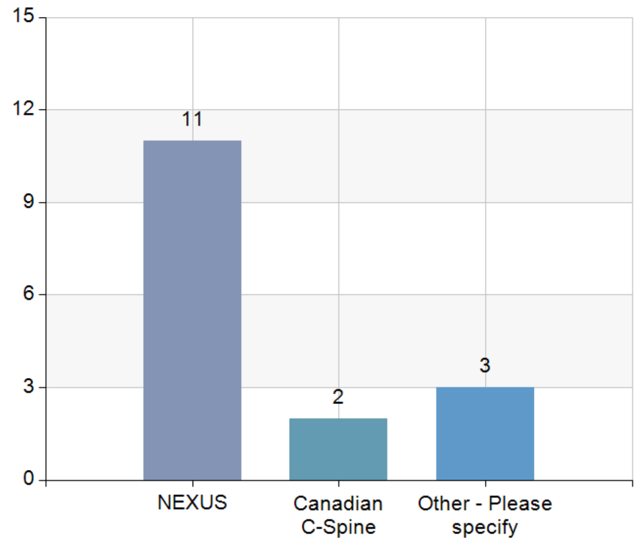

| **Other - Please specify** |
|---|
| combination of Nexus and Canadian C- spine - check Medicinska riktlinjer för ambulanssjukvården i Region Stockholm |
| NBV - spinal stabilisering |

• What is the primary referral center for a patient with suspected isolated SCI?

| Namn | Antal | % |
|---|---|---|
| Nearest local hospital | 3 | 14,3 |
| Nearest center with spine surgery service | 1 | 4,8 |
| University hospital | 15 | 71,4 |
| Other - Please specify | 2 | 9,5 |
| **Total** | **21** | **100** |

| Svarsfrekvens |
|---|
| 100% (21/21) |

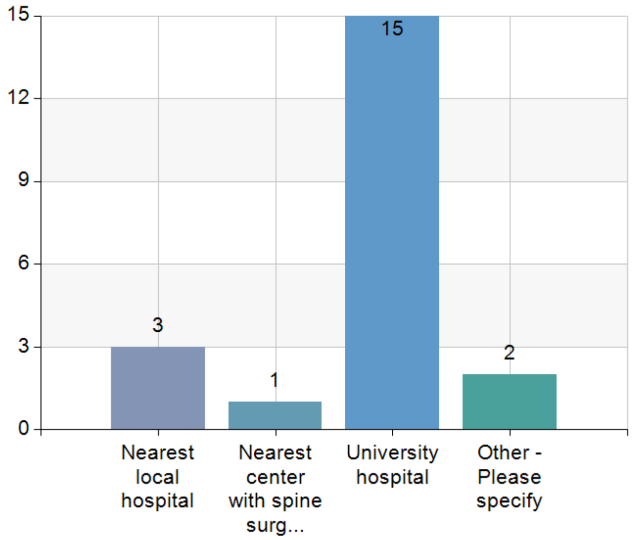


| **Other - Please specify** |
|---|
| Depends on trauma mechanism. Trauma center Karolinska Solna if induced by trauma |
| Nearest hospital with Trauma care |

• Do you have any comments related to the questions?

| |
|---|
| Registred nurses are mandatory in ambulances in Sweden. We dont have paramedics, ambulanssjukvårdare can be part of the team. In Region Stockholm Intubations are performed by Akutläkarbil anestesiologist, anestiology nurse or Emergency physician. |
| We have made guidelines for pre-hospital spine-stabilisation 2019. Vacuum mattress is the mode of stabilisation under transport. Use of hard-collar is less than before, but is not abandoned fully. |
| No |
| Backboards are allowed / used, if the prehospital phase won't exceed 30 min. |
| Some specialist nurses intubate. al staf are trained to secure airway with laryngeal mask |
| Backboard sometimes used for evacuation from accident sceene. Vacuum madrass for transport. |

• In the pre-hospital setting, do you have a specific blood pressure target in patients with suspected SCI?

| Namn | Antal | % |
|---|---|---|
| Yes | 12 | 57,1 |
| No | 9 | 42,9 |
| **Total** | **21** | **100** |

| Svarsfrekvens |
|---|
| 100% (21/21) |

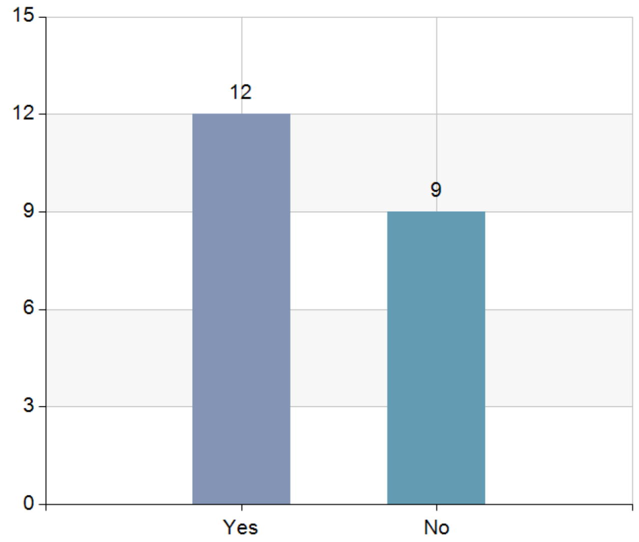


• If so, how is the blood pressure maintained within the target range?

| Namn | Antal | % |
|---|---|---|
| Volume expansion | 9 | 75 |
| Vasoactive drugs | 9 | 75 |
| **Total** | **18** | **150** |

| Svarsfrekvens |
|---|
| 57,1% (12/21) |

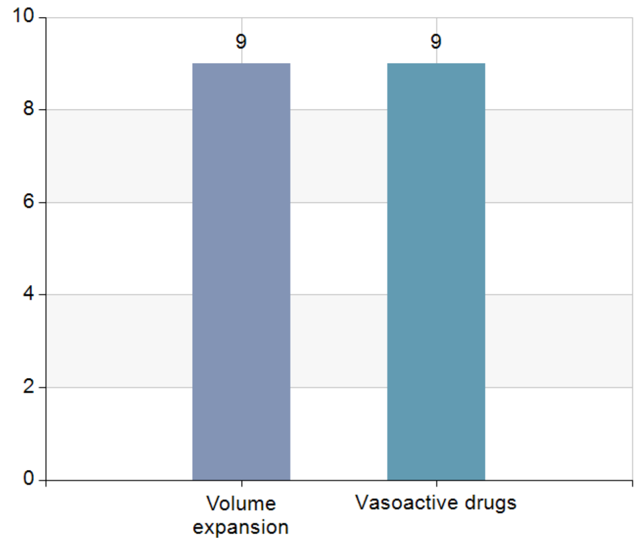


• If needed, what volume expansion is used in patients with suspected SCI?

| Namn | Antal | % |
|---|---|---|
| Crystalloids | 7 | 77,8 |
| Plasma | 0 | 0 |
| Albumin | 0 | 0 |
| Other - Please specify | 2 | 22,2 |
| **Total** | **9** | **100** |

| Svarsfrekvens |
|---|
| 42,9% (9/21) |

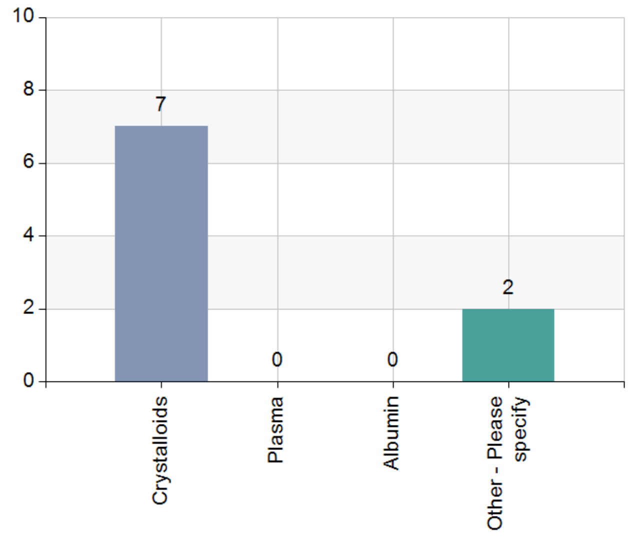

| Other - Please specify |
|---|
| depends on the injuries, if we suspect bleeding, we give blood&plasma, if spinal shock, we use noradrenaline |
| Full blood |

• If needed, which vasoactive drugs are used to maintain or elevate the blood pressure in patients with suspected SCI?

| Namn | Antal | % |
|---|---|---|
| Adrenaline | 1 | 11,1 |
| Noradrenaline | 9 | 100 |
| Dobutamine | 0 | 0 |
| Dopamine | 0 | 0 |
| Phenylephrine | 3 | 33,3 |
| Ephedrine | 4 | 44,4 |
| **Total** | **17** | **188,9** |

| Svarsfrekvens |
|---|
| 42,9% (9/21) |

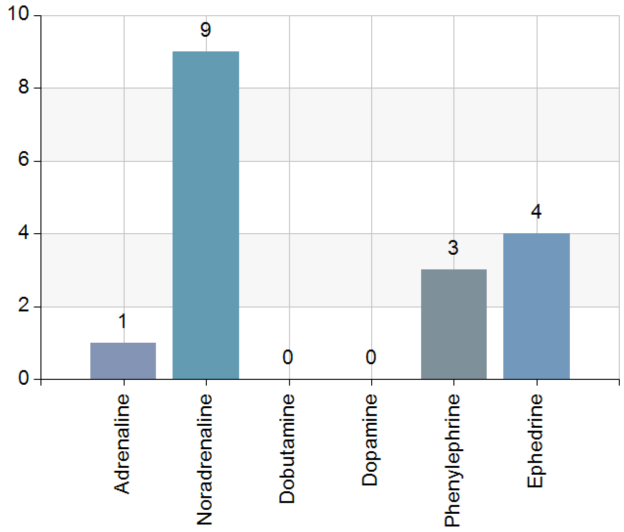


• In the pre-hospital setting, is MAP (mean arterial pressure) routinely measured in patients with suspected SCI?

| Namn | Antal | % |
|---|---|---|
| Most often | 11 | 52,4 |
| Sometimes | 5 | 23,8 |
| Seldom | 2 | 9,5 |
| Never | 3 | 14,3 |
| **Total** | **21** | **100** |

| Svarsfrekvens |
|---|
| 100% (21/21) |

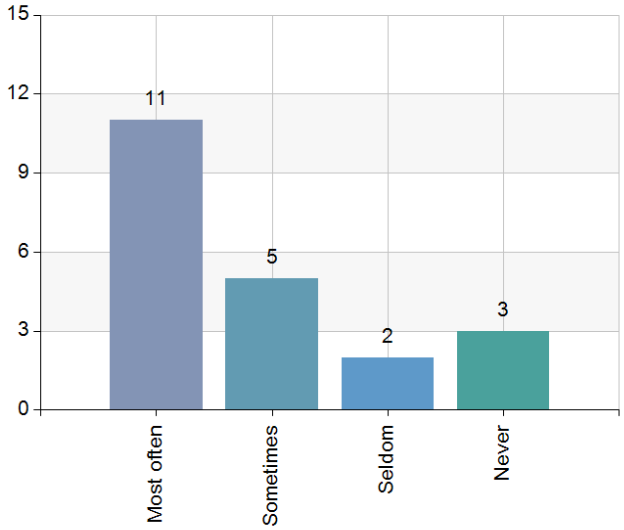


• Do you have any comments related to the questions?

| |
|---|
| Arterial lines placed prehospitally when necessary. |
| No specific blood pressure target for SCI, but there is a general target >90mmHg<br>MAP is available from the device, but not measured routinely. |
| Pre-hospital doctor-teams (HEMS) use MAP routinely, paramedics follow usually systolic pressures |
| We apply invasiv BT-mesure and target MAP > 80-85. May be a little volume before norepinephrine. (plasma 200 ml) |
| Isoleret sci hedder det MAP >80 ellers er det permissiv hypotension hvis der er andre organskader |

- Is tranexamic acid routinely administered in patients with suspected SCI?

| Namn | Antal | % |
|---|---|---|
| Yes | 5 | 23,8 |
| No | 16 | 76,2 |
| **Total** | **21** | **100** |

| Svarsfrekvens |
|---|
| 100% (21/21) |

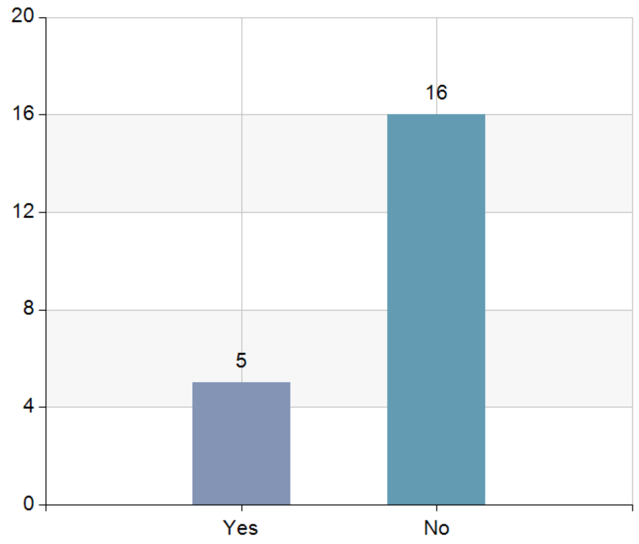


- Are high-dose steroids routinely administered to patients (i.v. or orally)?

| Namn | Antal | % |
|---|---|---|
| Yes, routinely | 0 | 0 |
| Yes, sometimes | 1 | 4,8 |
| No | 20 | 95,2 |
| **Total** | **21** | **100** |

| Svarsfrekvens |
|---|
| 100% (21/21) |

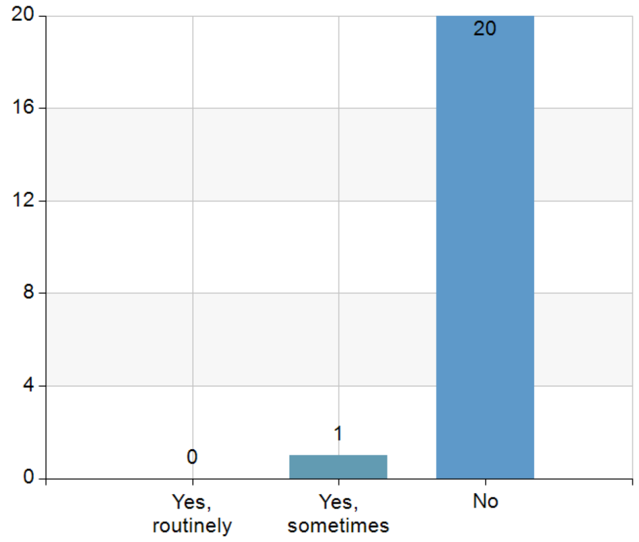


- Do you have any comments related to the questions?

| |
|---|
| TXA Is given if massive bleeding, ordered from Prehospital Doctor. AKutläkarbil. |
| To the above question: Steroids are not given pre-hospitally |
| Tranexamic acid is routinely administered to severely injured patients. Hence, it is administered to SCI patients also, as SCI patients are usually severely injured. |
| Answers primarily for the prehospital phase of SCI although I am familiar with the in-hospital / ICU treatment as well. |

• If you have finished answering the questions, please select "SUBMIT" below and then continue to the next page to submit your answers. Please note that this will lock and submit the survey and no further changes will be possible. • If you need more time to take the survey, you can continue at a later time. To do so, you can click the pause button at the bottom of the page. You can then resume taking the survey by following the link in the invitation e-mail that was sent to you earlier.

| Namn | Antal | % |
|---|---|---|
| SUBMIT | 19 | 100 |
| **Total** | **19** | **100** |

| Svarsfrekvens |
|---|
| 100% (19/19) |

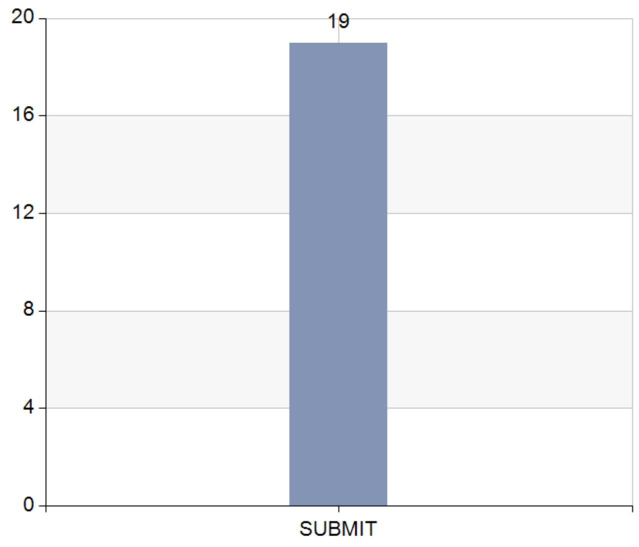


• Does your department use high-dose steroid infusion in patients with SCI (Spinal Cord Injury)?

| Namn | Antal | % |
|---|---|---|
| Yes, always | 0 | 0 |
| Yes, sometimes | 6 | 23,1 |
| No, never | 20 | 76,9 |
| **Total** | **26** | **100** |

| Svarsfrekvens |
|---|
| 100% (26/26) |

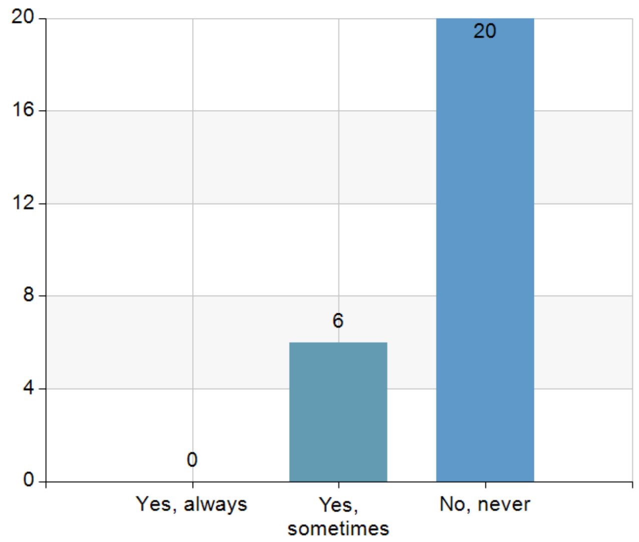


• Does your department administer other putative neuroprotective agents?

| Namn | Antal | % |
|---|---|---|
| Yes - Please specify | 0 | 0 |
| No | 26 | 100 |
| **Total** | **26** | **100** |

| Svarsfrekvens |
|---|
| 100% (26/26) |

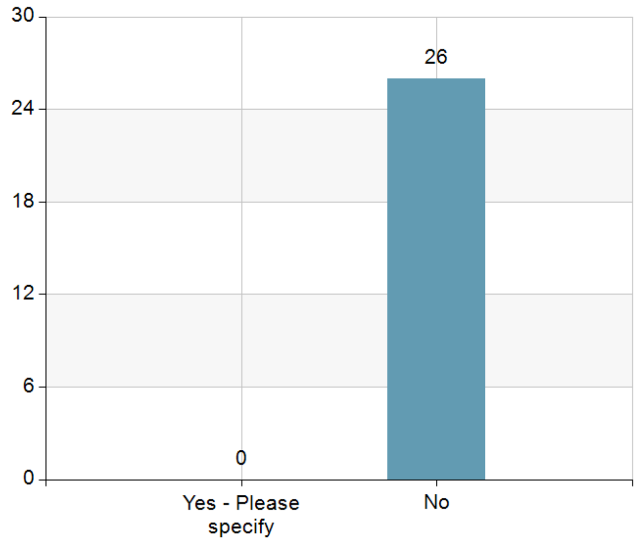


• Does your department apply lumbar drains to improve spinal perfusion?

| Namn | Antal | % |
|---|---|---|
| Yes, always | 0 | 0 |
| Yes, sometimes | 14 | 53,8 |
| No, never | 12 | 46,2 |
| **Total** | **26** | **100** |

| Svarsfrekvens |
|---|
| 100% (26/26) |

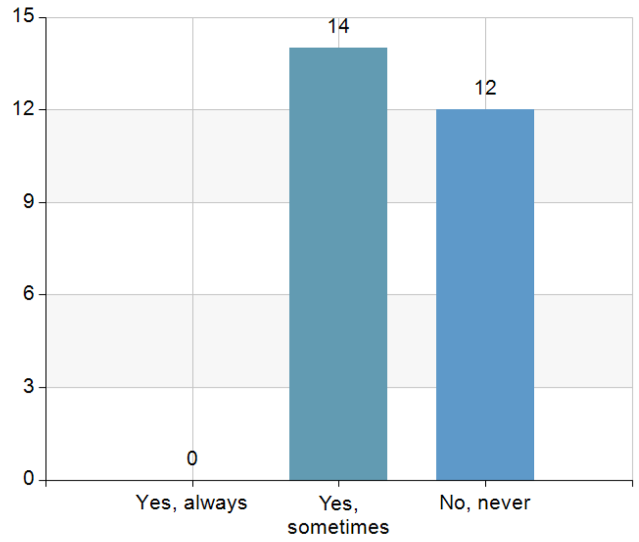


• In your department, is fever considered to negatively affect neurological outcome in patients with SCI?

| Namn | Antal | % |
|---|---|---|
| Yes | 20 | 76,9 |
| No | 6 | 23,1 |
| **Total** | **26** | **100** |

| Svarsfrekvens |
|---|
| 100% (26/26) |

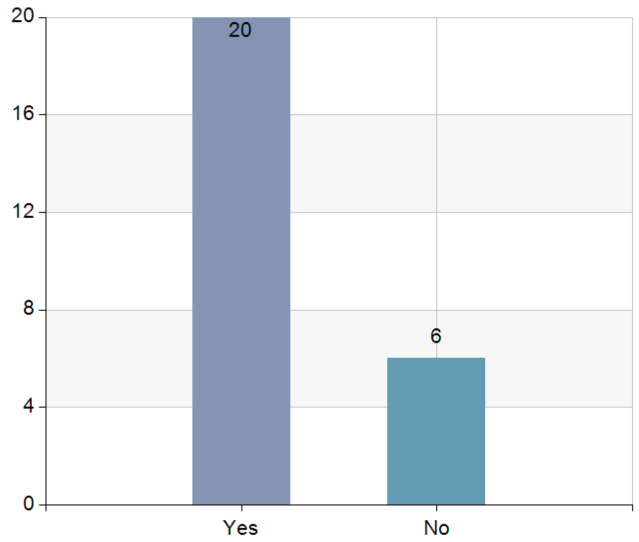

• If so, at what temperature threshold would treatment start for fever?

| Namn | Antal | % |
|---|---|---|
| 37.5 - 38 °C | 4 | 20 |
| 38 - 38.5 °C | 12 | 60 |
| 38.5 - 39 °C | 3 | 15 |
| >39 °C | 1 | 5 |
| Not sure | 0 | 0 |
| **Total** | **20** | **100** |

| Svarsfrekvens |
|---|
| 76,9% (20/26) |

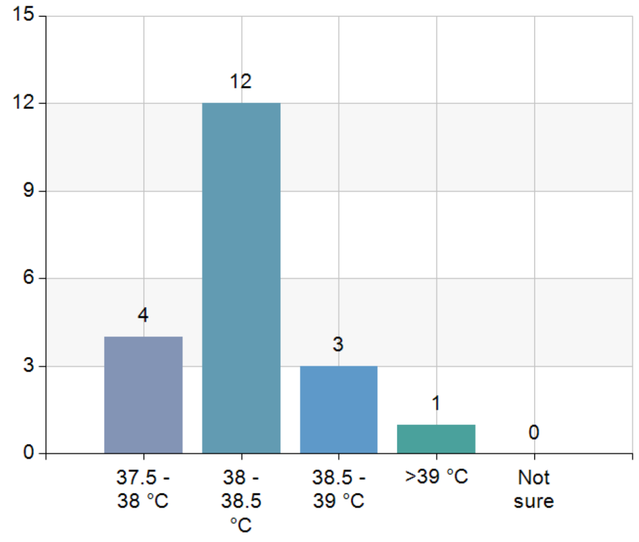


• Is a lower threshold level of hemoglobin used?

| Namn | Antal | % |
|---|---|---|
| Yes | 10 | 38,5 |
| No | 16 | 61,5 |
| **Total** | **26** | **100** |

| Svarsfrekvens |
|---|
| 100% (26/26) |

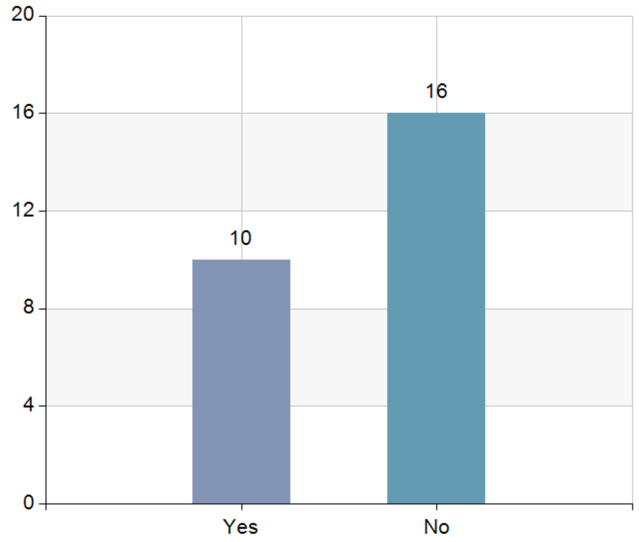


• If so, what threshold is used (g/L)?

| Namn | Antal | % |
|---|---|---|
| >70 | 3 | 30 |
| >80 | 2 | 20 |
| >90 | 4 | 40 |
| >100 | 1 | 10 |
| >110 | 0 | 0 |
| >120 | 0 | 0 |
| Other - Please specify | 0 | 0 |
| **Total** | **10** | **100** |

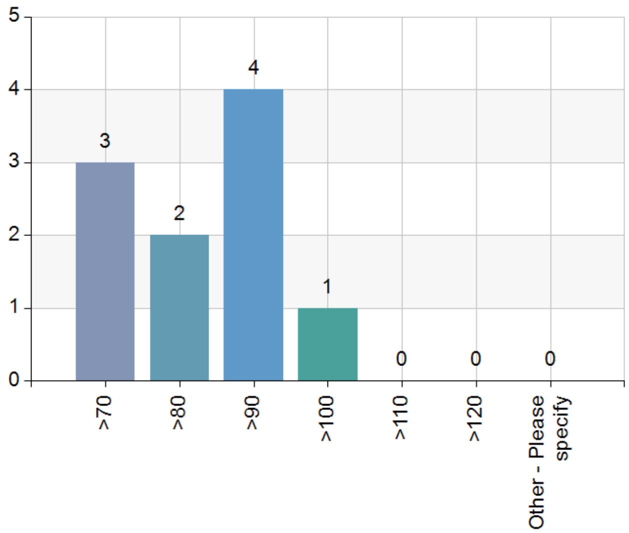


| Svarsfrekvens |
|---|
| 38,5% (10/26) |

- Do you have any comments related to the questions?

| |
|---|
| We treat SCI and TBI (traumatic brain injury) very similar - by applying normal Hb levels, normal albumine levels, normal sodium levels, normal ABGs... MAP>70 mmHg. |
| It would be helpful if you had an alternative such as not applicable. In my region we don’t receive this patients primarily they are transferred to the trauma centre directly from the scene of accident. |
| We do not have any written guidelines regarding ICU-treatment in patients with SCI. |

- Does your department use a specific MAP (Mean Arterial Pressure) protocol?

| Namn | Antal | % |
|---|---|---|
| Yes, always | 17 | 65,4 |
| Yes, sometimes | 6 | 23,1 |
| No, never | 3 | 11,5 |
| **Total** | **26** | **100** |

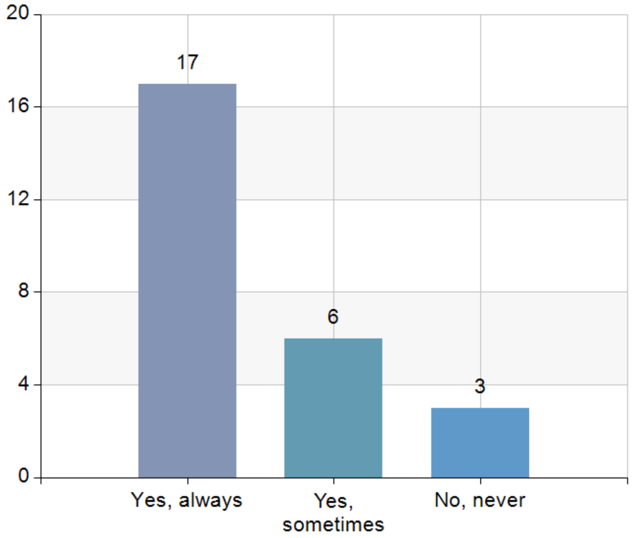


| Svarsfrekvens |
|---|
| 100% (26/26) |

- If so, what is the target value for MAP?

| Namn | Antal | % |
|---|---|---|
| >80 mmHg | 12 | 54,5 |
| >85 mmHg | 7 | 31,8 |
| >90 mmHg | 3 | 13,6 |
| >95 mmHg | 0 | 0 |
| **Total** | **22** | **100** |

| Svarsfrekvens |
|---|
| 84,6% (22/26) |

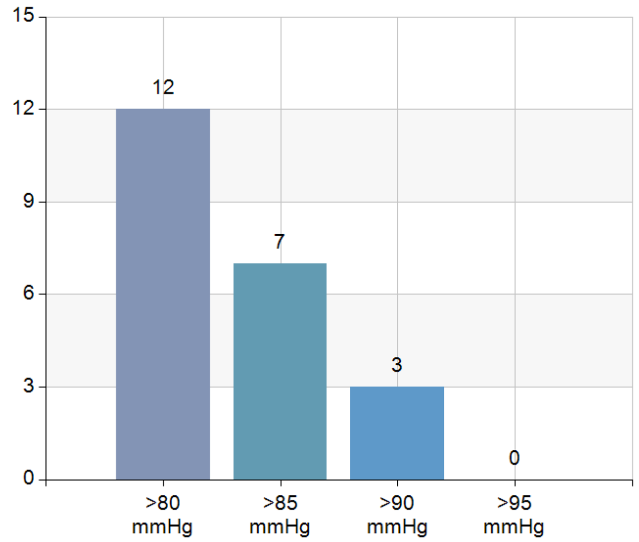


• For how long is the target value appropriate?

| Namn | Antal | % |
|---|---|---|
| 1-3 days | 8 | 34,8 |
| 4-7 days | 11 | 47,8 |
| >7 days | 4 | 17,4 |
| **Total** | **23** | **100** |

| Svarsfrekvens |
|---|
| 88,5% (23/26) |

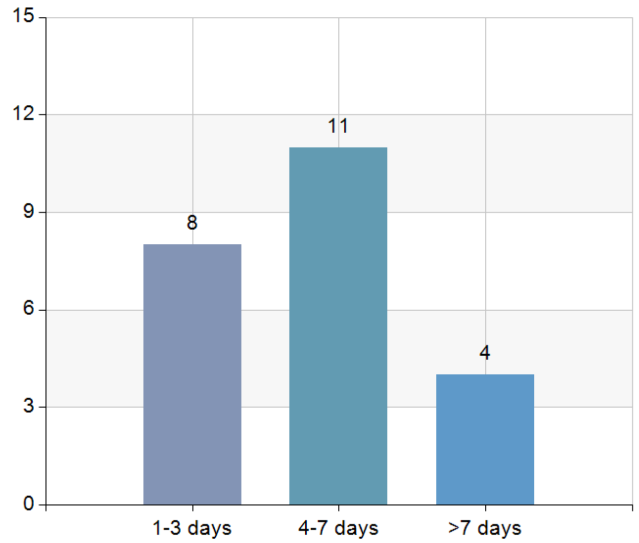


• What is the choice of drug(s) to maintain adequate MAP?

| Namn | Antal | % |
|---|---|---|
| Adrenaline | 0 | 0 |
| Noradrenaline | 22 | 95,7 |
| Dobutamine | 3 | 13 |
| Dopamine | 0 | 0 |
| Phenylephrine | 0 | 0 |
| Ephedrine | 0 | 0 |
| **Total** | **25** | **108,7** |

| Svarsfrekvens |
|---|
| 88,5% (23/26) |

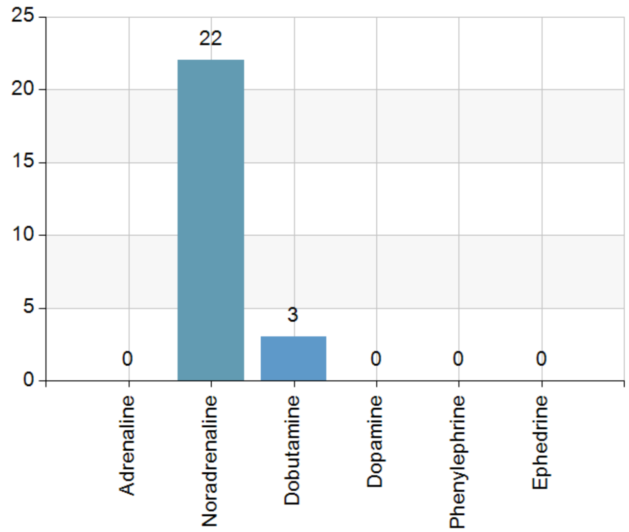


• If needed, what volume expander is used?

| Namn | Antal | % |
|---|---|---|
| Crystalloids | 16 | 61,5 |
| Plasma | 0 | 0 |
| Albumin | 10 | 38,5 |
| Other colloids - Please specify | 0 | 0 |
| **Total** | **26** | **100** |

| Svarsfrekvens |
|---|
| 100% (26/26) |

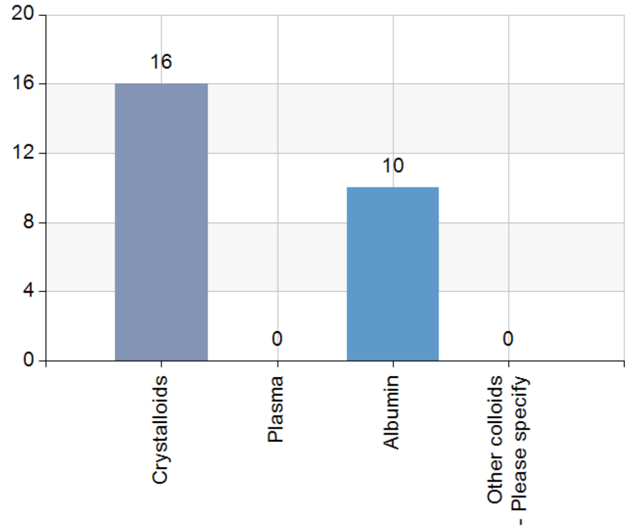


• Do you have any comments related to the questions?

| |
|---|
| MAP>70. After patients kan swallow - pills with etilefrin is given if needed. |
| You should allow multiple answers |
| Mixed crystalloids and albumin |
| MAP for 7 days |
| We use both crystalloids and albumin depending on for example fluidbalance. |

• Post-operatively, at what time is active physical rehabilitation initiated (including in-bed exercises)?

| Namn | Antal | % |
|---|---|---|
| 1-3 days | 21 | 84 |
| 4-7 days | 3 | 12 |
| >7 days | 1 | 4 |
| **Total** | **25** | **100** |

| Svarsfrekvens |
|---|
| 96,2% (25/26) |

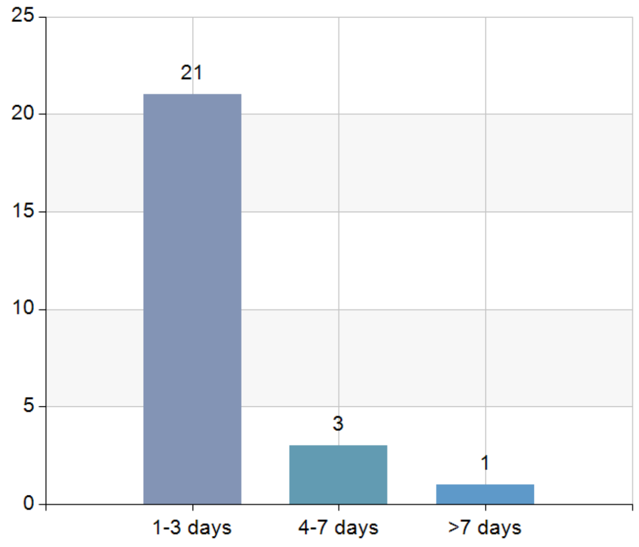


• Who performs mobilization of patients with SCI?

| Namn | Antal | % |
|---|---|---|
| ICU nurses | 19 | 73,1 |
| Physical therapists | 26 | 100 |
| ICU doctors | 1 | 3,8 |
| Other ICU staff | 5 | 19,2 |
| Other - Please specify | 0 | 0 |
| **Total** | **51** | **196,2** |

| Svarsfrekvens |
|---|
| 100% (26/26) |

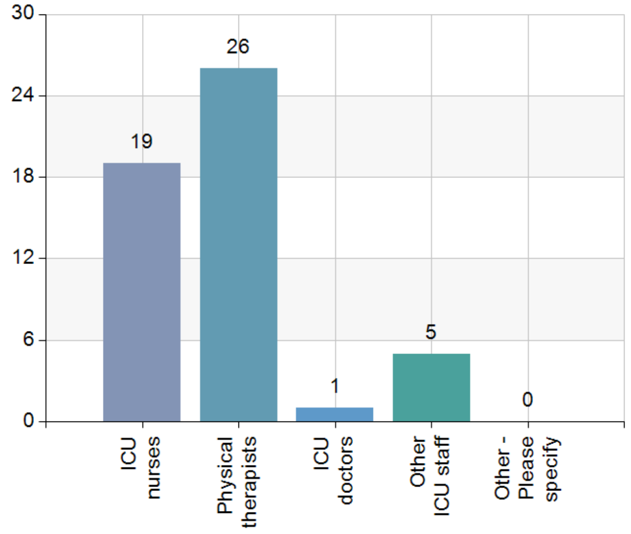


• What is the frequency of physical therapy in SCI patients?

| Namn | Antal | % |
|---|---|---|
| Several times daily | 16 | 61,5 |
| Once daily | 10 | 38,5 |
| Occasionally | 0 | 0 |
| **Total** | **26** | **100** |

| Svarsfrekvens |
|---|
| 100% (26/26) |

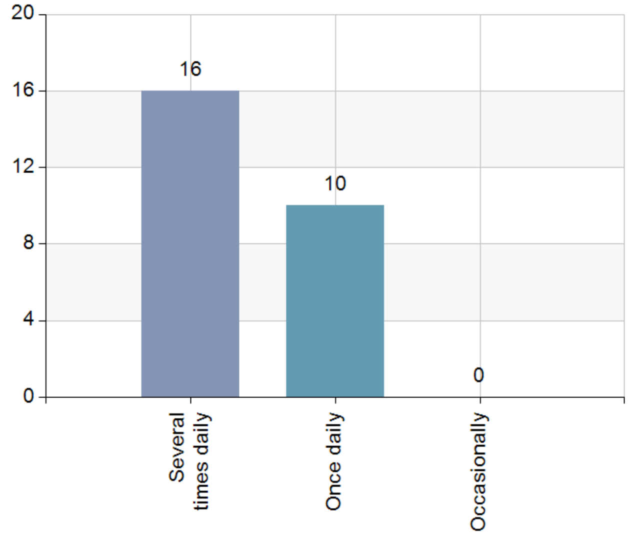

• Do you have any comments related to the questions?

| |
|---|
| Patients are put in a supine position if breathing is affected - and always moved from side to side frequently to avoid decubitus.<br>Patients may sit up when the blood pressure allows it, and always gradually and very careful. |
| Again you should have a alternative not applicable |
| No particular local programme for physical therapy aimed at SCI-patients in particular exist at our ICU. This is the general approach to a longstayer in the ICU. |

• Provided no other injuries, at what time is thromboprophylactic treatment initiated after spinal surgery?

| Namn | Antal | % |
|---|---|---|
| <24 hours | 8 | 32 |
| 24-48 hours | 17 | 68 |
| 48-72 hours | 0 | 0 |
| >72 hours | 0 | 0 |
| **Total** | **25** | **100** |

| Svarsfrekvens |
|---|
| 96,2% (25/26) |

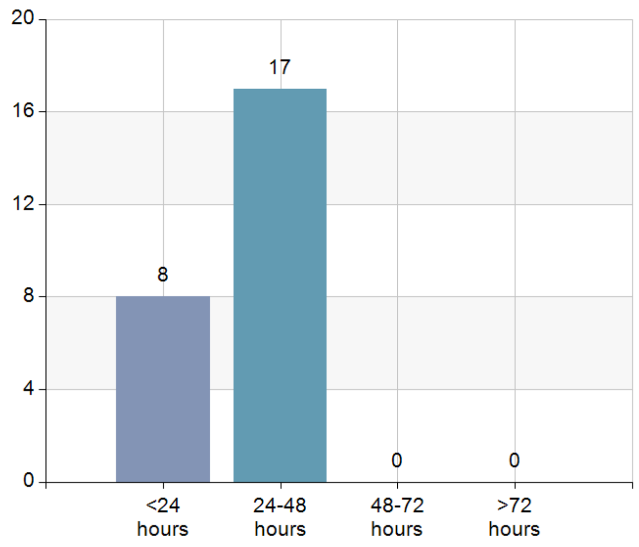


• In patients with SCI and neurological deficits affecting the degree of mobilization - for how long is thromboprophylactic treatment considered necessary?

| Namn | Antal | % |
|---|---|---|
| <2 weeks | 1 | 4,8 |
| 2-4 weeks | 9 | 42,9 |
| 1-3 months | 10 | 47,6 |
| >3 months | 1 | 4,8 |
| **Total** | **21** | **100** |

| Svarsfrekvens |
|---|
| 80,8% (21/26) |

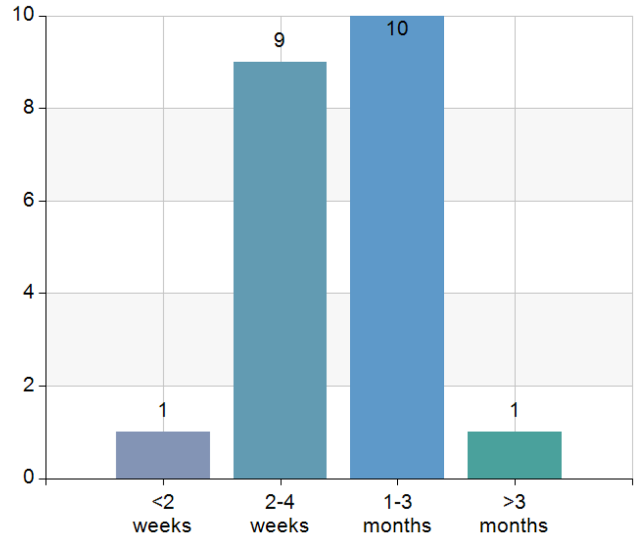


• Are compression stockings part of the thromboprophylactic protocol?

| Namn | Antal | % |
|---|---|---|
| Yes, always | 10 | 40 |
| Yes, sometimes | 14 | 56 |
| No, never | 1 | 4 |
| **Total** | **25** | **100** |

| Svarsfrekvens |
|---|
| 96,2% (25/26) |

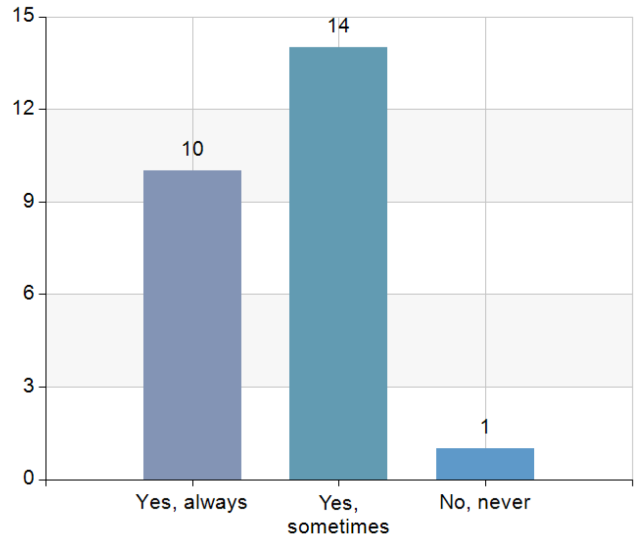


• What thromboprophylactic treatment is the primary choice of your department?

| Namn | Antal | % |
|---|---|---|
| Low-molecular-weight heparin | 25 | 96,2 |
| Heparin infusion | 0 | 0 |
| Direct oral anticoagulant | 0 | 0 |
| Warfarin | 0 | 0 |
| Other - Please specify | 1 | 3,8 |
| **Total** | **26** | **100** |

| Svarsfrekvens |
|---|
| 100% (26/26) |

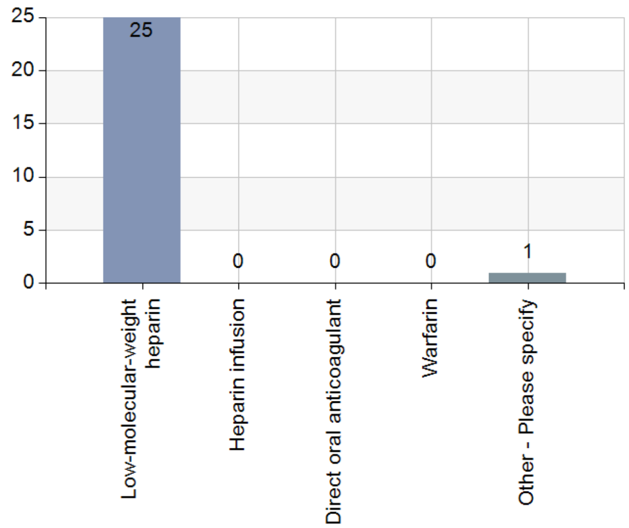


| **Other - Please specify** |
|---|
| Mechanical IPC |

• Do you have any comments related to the questions?

| |
|---|
| Se the comments before |
| DVT-prophylaxis is ordered by the orthopaedic surgeon or in dialogue whith them, particulary in the early phases of the course. |
| I am not sure about the answers. The orthopedic surgeons is responsible for antikoagulant treatment. |
| not sure of question 4.2 |

• At what time is enteral feeds initiated post-operative?

| Namn | Antal | % |
|---|---|---|
| <24 hours | 13 | 50 |
| 24-48 hours | 12 | 46,2 |
| >48 hours | 1 | 3,8 |
| **Total** | **26** | **100** |

| Svarsfrekvens |
|---|
| 100% (26/26) |

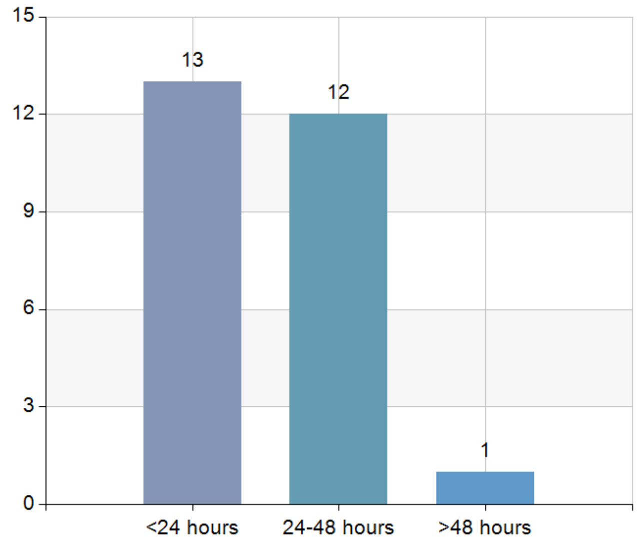


- Is a standardized regimen applied for bowel emptying to mitigate constipation?

| Namn | Antal | % |
|---|---|---|
| Yes, always | 20 | 76,9 |
| Yes, sometimes | 5 | 19,2 |
| No, never | 1 | 3,8 |
| **Total** | **26** | **100** |

| Svarsfrekvens |
|---|
| 100% (26/26) |

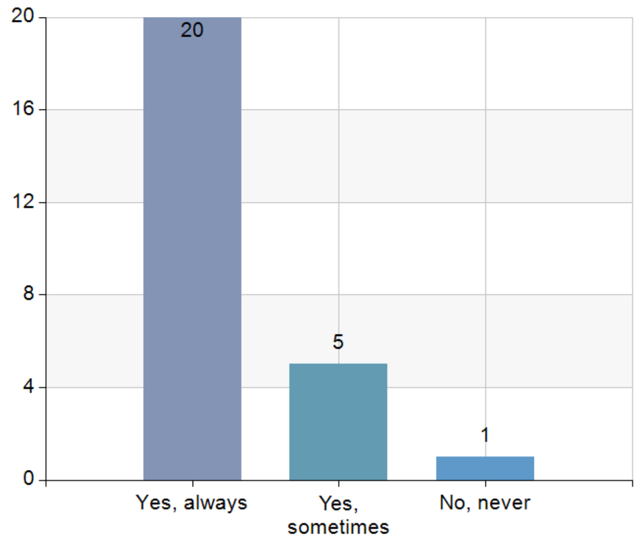


- Are GABAergic agents routinely applied in the ICU as part of the analgesic regimen?

| Namn | Antal | % |
|---|---|---|
| Yes, always | 1 | 3,8 |
| Yes, sometimes | 21 | 80,8 |
| No, never | 4 | 15,4 |
| **Total** | **26** | **100** |

| Svarsfrekvens |
|---|
| 100% (26/26) |

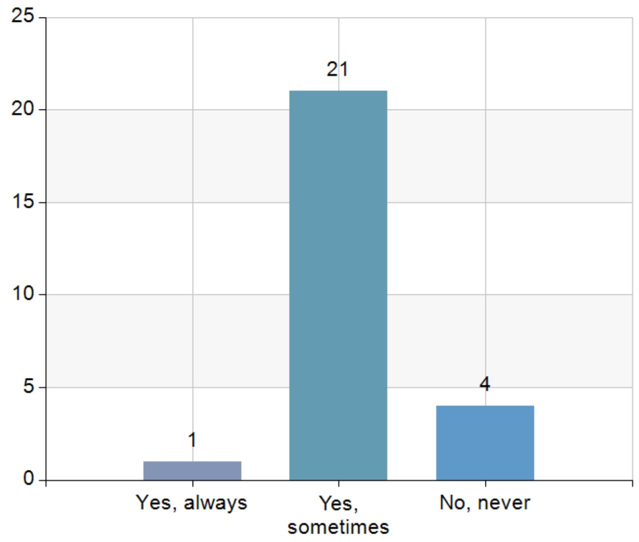

• Do you have any comments related to the questions?

| |
|---|
| When indicated |
| All these things are indivualized in our ICU, and we do not have a particular guideline for SCI-patients. |

• If you have finished answering the questions, please select "SUBMIT" below and then continue to the next page to submit your answers. Please note that this will lock and submit the survey and no further changes will be possible. • If you need more time to take the survey, you can continue at a later time. To do so, you can click the pause button at the bottom of the page. You can then resume taking the survey by following the link in the invitation e-mail that was sent to you earlier.

| Namn | Antal | % |
|---|---|---|
| SUBMIT | 14 | 100 |
| **Total** | **14** | **100** |

| Svarsfrekvens |
|---|
| 100% (14/14) |

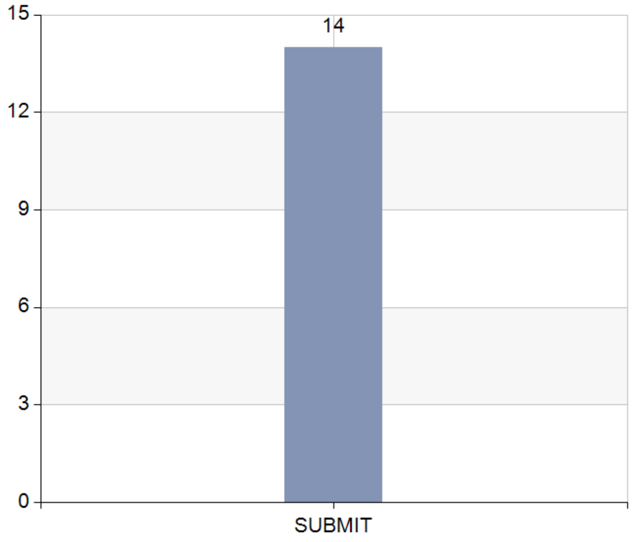


• Upon arrival at your hospital, which medical specialty performs the initial neurological assessment of a patient with suspected SCI (Spinal Cord Injury)?

| Namn | Antal | % |
|---|---|---|
| Trauma surgeon | 23 | 65,7 |
| Neurosurgeon | 14 | 40 |
| General surgeon | 5 | 14,3 |
| Orthopedic surgeon | 20 | 57,1 |
| Neurologist | 0 | 0 |
| Other - Please specify | 3 | 8,6 |
| **Total** | **65** | **185,7** |

| Svarsfrekvens |
|---|
| 100% (35/35) |

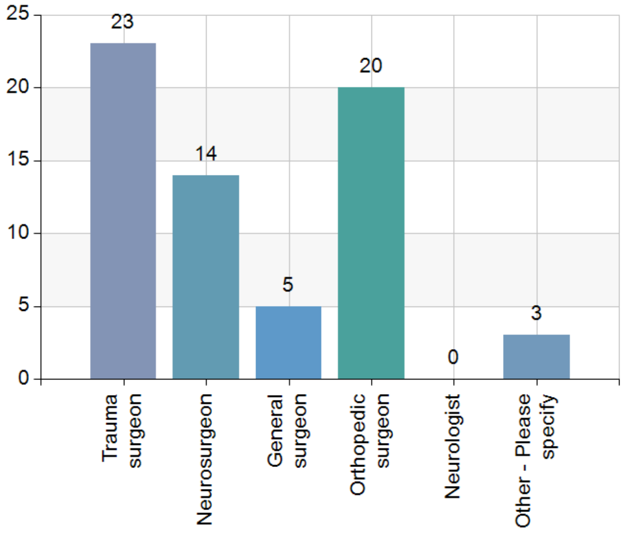

| Other - Please specify |
|---|
| ER specialist |
| ”Akutläkare” |
| Ortho spine surgeon |

• Is a standardized form routinely used and registered to assess the neurological status of such patients?

| Namn | Antal | % |
|---|---|---|
| Yes, always | 13 | 37,1 |
| Yes, sometimes | 13 | 37,1 |
| No, never | 9 | 25,7 |
| **Total** | **35** | **100** |

| Svarsfrekvens |
|---|
| 100% (35/35) |

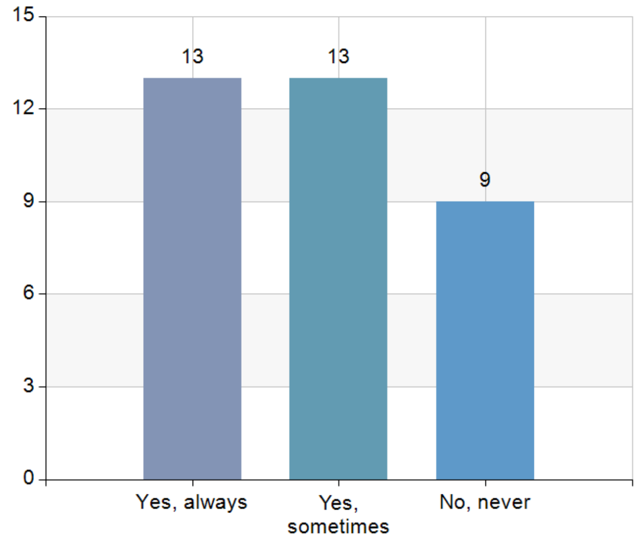


• Do you have any comments related to the questions?

| |
|---|
| Neurological status is assessed by a specialized team (physician + physioterapeut ) from the Dept of Rehabilitionmedicine within 1-3 days after arrival |
| In the very beginning there are no forms to be filled up. After the surgical treatment, there are forms like ASIA. |
| We have no standardized protocol for this since quite some time |
| In a trauma setting there is no room for complete AISA scoore. We have adapted short version AISA, and AO spine classification N0-NX . The traunma surgeon are requested to specify muscle functions and Sphincther tounus if possible. |

• In your department, are high-dose intravenous steroid injections routinely given to patients with suspected or known SCI?

| Namn | Antal | % |
|---|---|---|
| Yes, always | 0 | 0 |
| Yes, sometimes | 6 | 17,1 |
| No, never | 29 | 82,9 |
| **Total** | **35** | **100** |

| Svarsfrekvens |
|---|
| 100% (35/35) |

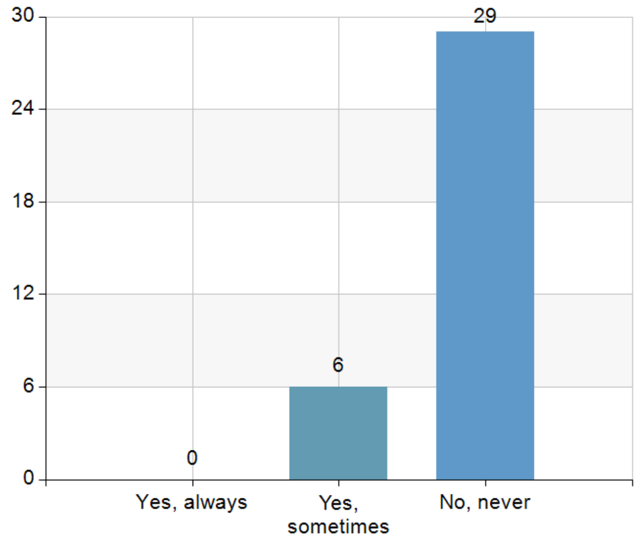


• If so, at what time interval after injury is steroid infusion started? [text]

| Namn | Antal | % |
|---|---|---|
| <8 hours | 4 | 66,7 |
| <24 hours | 2 | 33,3 |
| <48 hours | 0 | 0 |
| >48 hours | 0 | 0 |
| **Total** | **6** | **100** |

| Svarsfrekvens |
|---|
| 17,1% (6/35) |

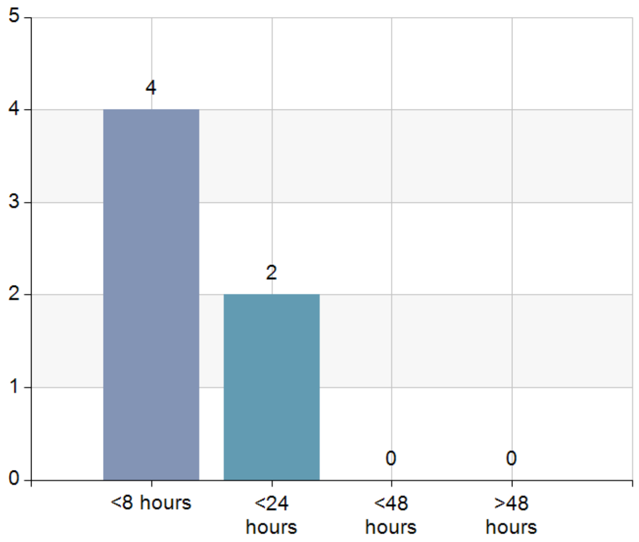


• If steroid treatment is routinely applied, for how long is this provided?

| Namn | Antal | % |
|---|---|---|
| <24 hours | 2 | 40 |
| 24<48 hours | 2 | 40 |
| >48 hours | 1 | 20 |
| **Total** | **5** | **100** |

| Svarsfrekvens |
|---|
| 14,3% (5/35) |

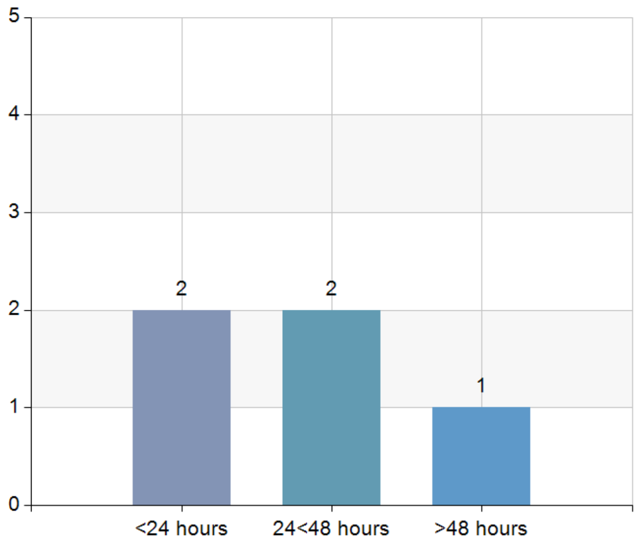

• Do you have any comments related to the questions?

| |
|---|
| It was used many years ago. |
| In metastatic disease we frequently use |
| Corticosteroid are used only on tumor patients. Never for acute fractures. |
| Steroids are removed from ATLS protocol. |

• Does your department have a specific MAP (Mean Arterial Pressure) target in patients with suspected SCI?

| Namn | Antal | % |
|---|---|---|
| Yes, always | 23 | 65,7 |
| Yes, sometimes | 9 | 25,7 |
| No, never | 3 | 8,6 |
| **Total** | **35** | **100** |

| Svarsfrekvens |
|---|
| 100% (35/35) |

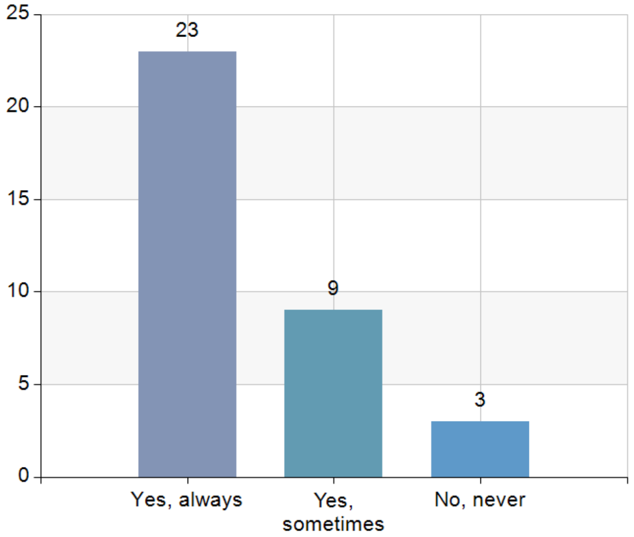


• If so, what is the MAP target?

| Namn | Antal | % |
|---|---|---|
| >80 mmHg | 13 | 40,6 |
| >85 mmHg | 15 | 46,9 |
| >90 mmHg | 4 | 12,5 |
| >95 mmHg | 0 | 0 |
| **Total** | **32** | **100** |

| Svarsfrekvens |
|---|
| 91,4% (32/35) |

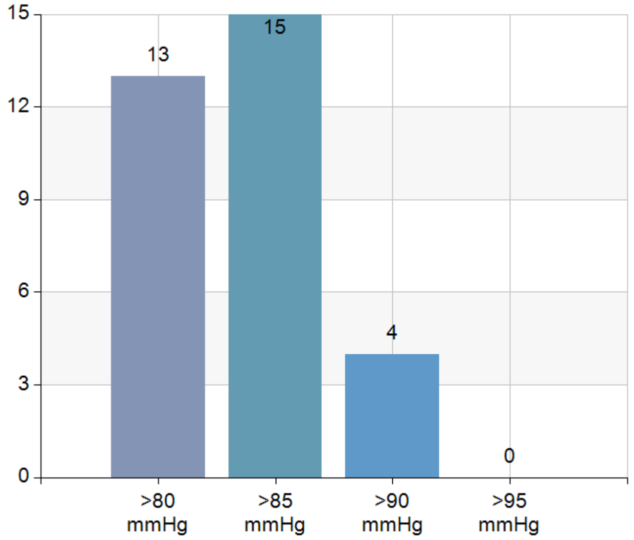


• If MAP is targeted, is invasive blood pressure (arterial) monitoring used?

| Namn | Antal | % |
|---|---|---|
| Yes, always | 28 | 87,5 |
| Yes, sometimes | 4 | 12,5 |
| No, never | 0 | 0 |
| **Total** | **32** | **100** |

| Svarsfrekvens |
|---|
| 91,4% (32/35) |

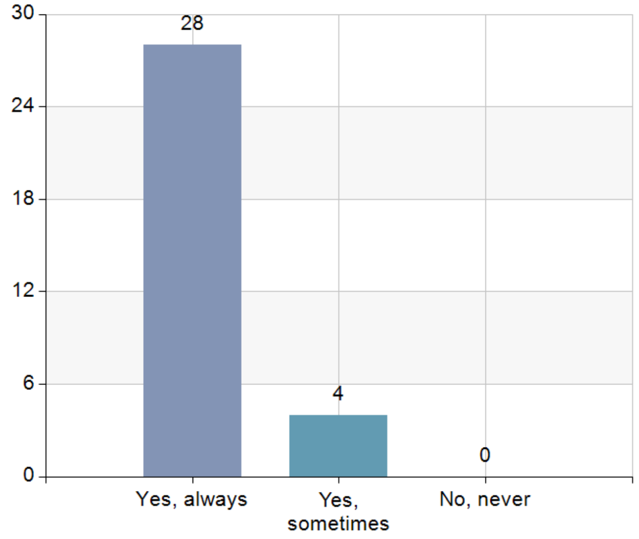


• What time interval is elevated MAP applied?

| Namn | Antal | % |
|---|---|---|
| 1-3 days | 15 | 46,9 |
| 4-7 days | 14 | 43,8 |
| >7 days | 3 | 9,4 |
| **Total** | **32** | **100** |

| Svarsfrekvens |
|---|
| 91,4% (32/35) |

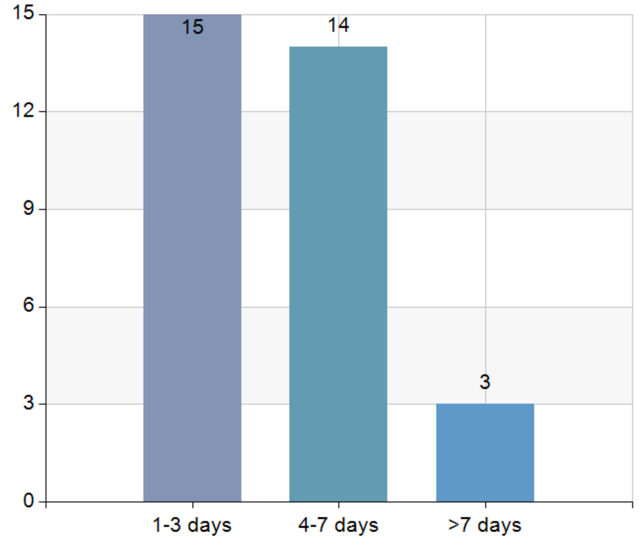


• Do you have any comments related to the questions?

| |
|---|
| Time interval after agreement between ICU anesthesiologist and orthopedic surgeon / neurosurgeon.<br>Most common 1-3 days and always until surgical treatment is performed, if this is planned. |
| This is very interesting issue to clarify! Waiting for your results... :-) |
| Not involved so I dont know |
| elevated MAP is applied for exactly 7 days post trauma |
| MAP> 80 if 16 years MAP>85. MAP targeted in CC, THx, not if injury is below cauda. |
| Elevated MAP not applied after decompressive surgery or closed reduction and MRI verified relief of compression. |

• Are spine X-rays routinely used as part of the initial radiological assessment?

| Namn | Antal | % |
|---|---|---|
| Yes, always | 9 | 25,7 |
| Yes, sometimes | 11 | 31,4 |
| No, never | 15 | 42,9 |
| **Total** | **35** | **100** |

| Svarsfrekvens |
|---|
| 100% (35/35) |

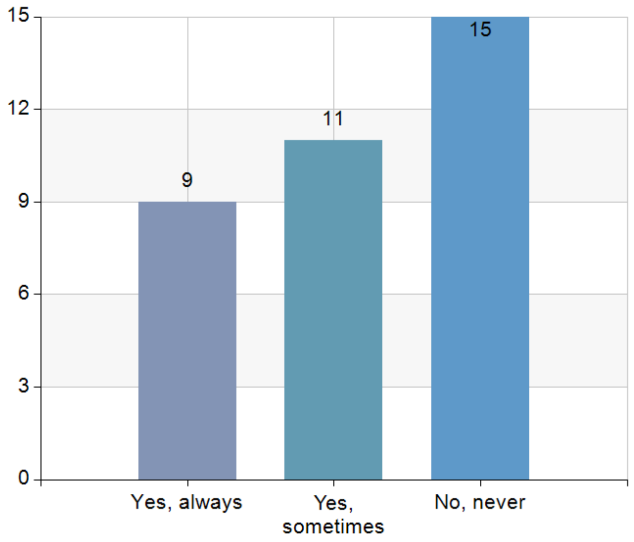


• Is MRI (Magnetic Resonance Imaging) available as a 24/7 service at your institution?

| Namn | Antal | % |
|---|---|---|
| Yes | 33 | 94,3 |
| No | 2 | 5,7 |
| **Total** | **35** | **100** |

| Svarsfrekvens |
|---|
| 100% (35/35) |

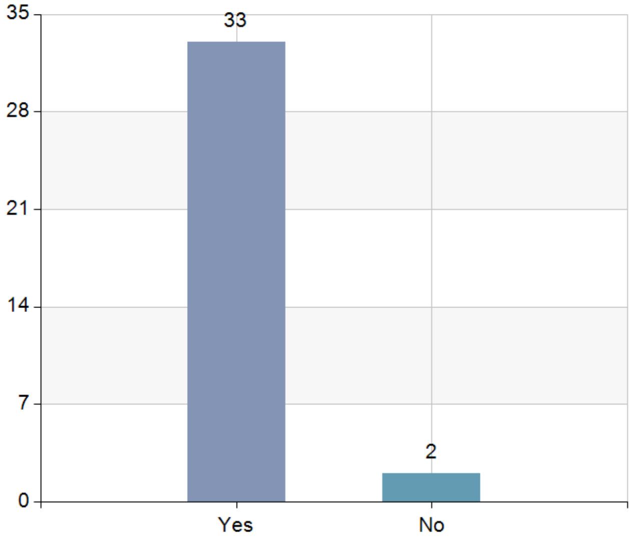


• Is MRI part of the acute (

| Namn | Antal | % |
|---|---|---|
| Yes, always | 29 | 82,9 |
| Yes, sometimes | 6 | 17,1 |
| No, never | 0 | 0 |
| **Total** | **35** | **100** |

| Svarsfrekvens |
|---|
| 100% (35/35) |

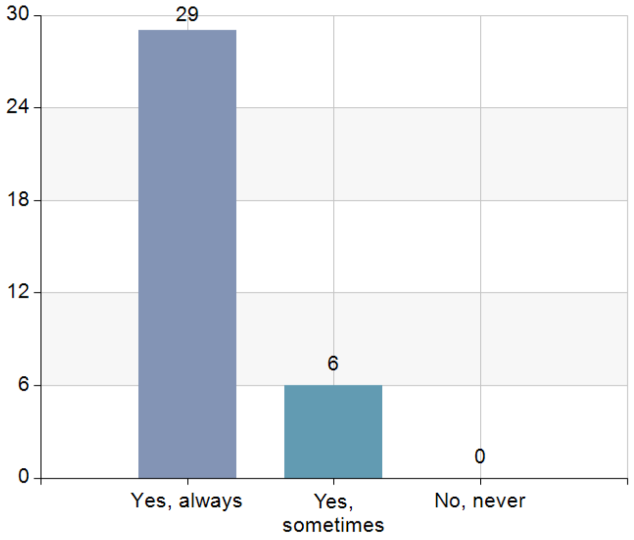

• Is MRI considered compulsory before surgery is performed in patients with suspected SCI? (Level of injury C1-L1)

| Namn | Antal | % |
|---|---|---|
| Yes, always | 23 | 65,7 |
| Yes, sometimes | 10 | 28,6 |
| No, never | 2 | 5,7 |
| **Total** | **35** | **100** |

| Svarsfrekvens |
|---|
| 100% (35/35) |

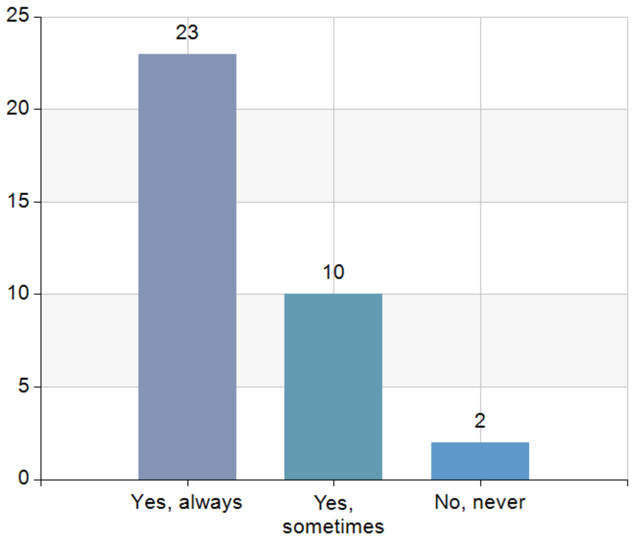


• Is an MRI of the brain part of the initial radiological assessment when cervical spinal cord injury is demonstrated?

| Namn | Antal | % |
|---|---|---|
| Yes, always | 2 | 6,1 |
| Yes, sometimes | 27 | 81,8 |
| No, never | 4 | 12,1 |
| **Total** | **33** | **100** |

| Svarsfrekvens |
|---|
| 94,3% (33/35) |

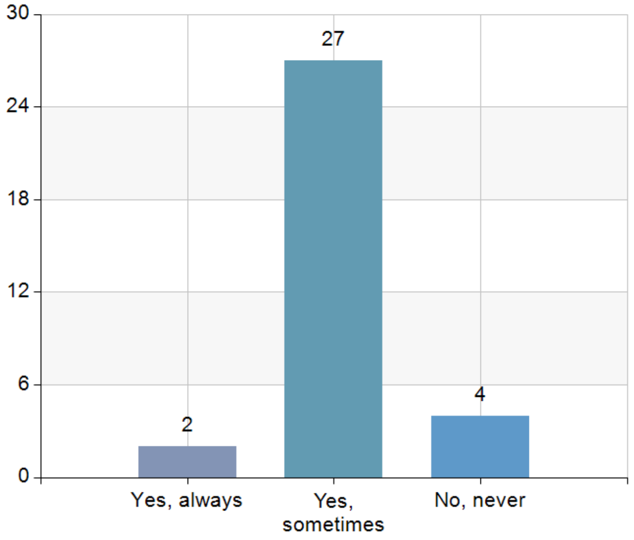


• Is a CT (Computed Tomography) angiogram of pre-vertebral arteries part of the radiological investigations when cervical spine injury is demonstrated?

| Namn | Antal | % |
|---|---|---|
| Yes, always | 11 | 33,3 |
| Yes, sometimes | 22 | 66,7 |
| No, never | 0 | 0 |
| **Total** | **33** | **100** |

| Svarsfrekvens |
|---|
| 94,3% (33/35) |

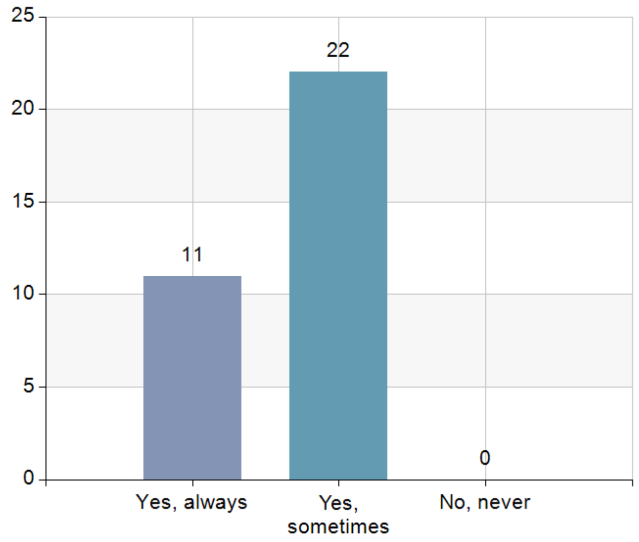


• Is a standard classification scheme routinely used for radiological evaluation of spine injuries?

| Namn | Antal | % |
|---|---|---|
| Yes, always | 8 | 24,2 |
| Yes, sometimes | 15 | 45,5 |
| No, never | 10 | 30,3 |
| **Total** | **33** | **100** |

| Svarsfrekvens |
|---|
| 94,3% (33/35) |

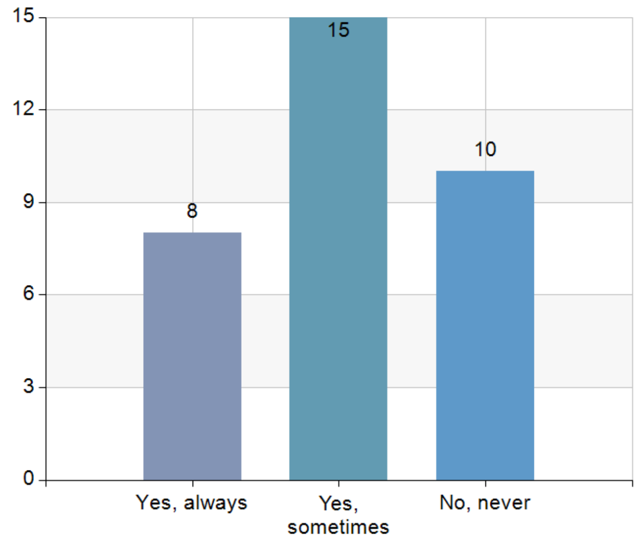


• If so, what classification system is most often used in your department?

| Namn | Antal | % |
|---|---|---|
| AO | 15 | 65,2 |
| SLICS | 3 | 13 |
| TLICS | 5 | 21,7 |
| Denise | 0 | 0 |
| Other - Please specify | 0 | 0 |
| **Total** | **23** | **100** |

| Svarsfrekvens |
|---|
| 65,7% (23/35) |

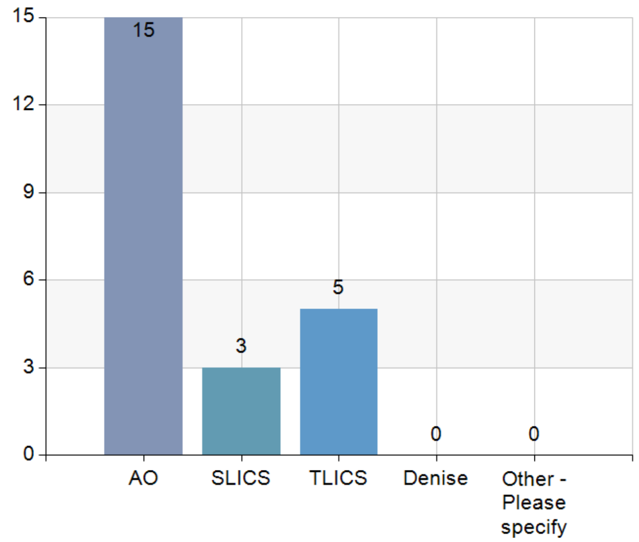

• Do you have any comments related to the questions?

| |
|---|
| Also TL-AOSIS And TLICS |
| I can’t answer the questions regarding treatment and radiologic examination of the cervical spine because evaluation and treatment of these injuries is performed by the neurosurgeons. |
| Should have 2 possibilities we use AO and SLICS |
| Minority of surgeon use AO |
| no spine x-rays are perfomerd but all patientens have done a primary Trauma-CT. |
| We always take CT complete spine if fracture, and therefor not always spine x ray |
| Spine X-rays indicated, but this means computed tomography, never conventional radiographs |

• Is skull traction used in patients with unstable C-spine injury?

| Namn | Antal | % |
|---|---|---|
| Yes, always | 2 | 6,1 |
| Yes, sometimes | 23 | 69,7 |
| No, never | 5 | 15,2 |
| Not applicable | 3 | 9,1 |
| **Total** | **33** | **100** |

| Svarsfrekvens |
|---|
| 94,3% (33/35) |

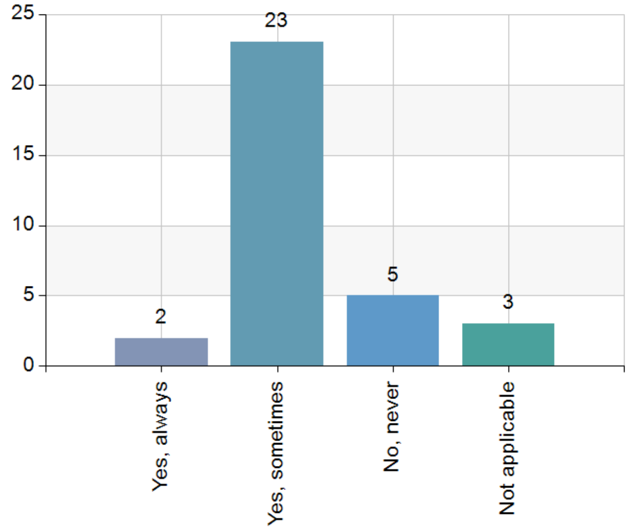


• What time frame does your department regard as early surgery for patients with SCI?

| Namn | Antal | % |
|---|---|---|
| <8 hours | 15 | 42,9 |
| <24 hours | 20 | 57,1 |
| <48 hours | 0 | 0 |
| 48-72 hours | 0 | 0 |
| **Total** | **35** | **100** |

| Svarsfrekvens |
|---|
| 100% (35/35) |

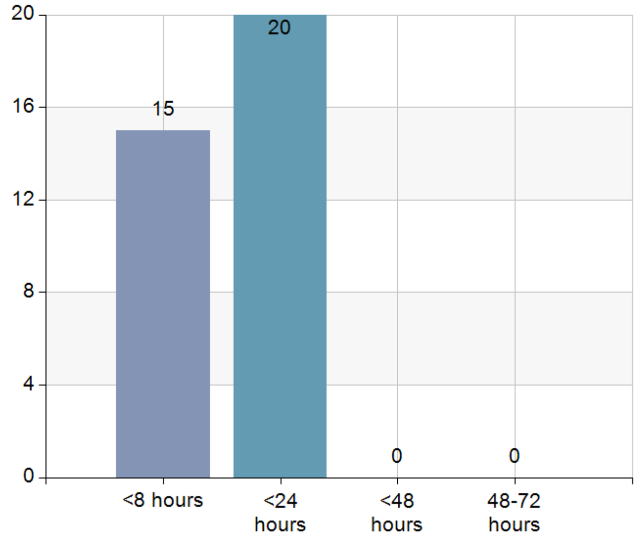

• Is timing of surgery depending on the neurological status (i.e., ASIA classification) of the patient?

| Namn | Antal | % |
|---|---|---|
| Yes, always | 22 | 64,7 |
| Yes, sometimes | 11 | 32,4 |
| No, never | 1 | 2,9 |
| **Total** | **34** | **100** |

| Svarsfrekvens |
|---|
| 97,1% (34/35) |

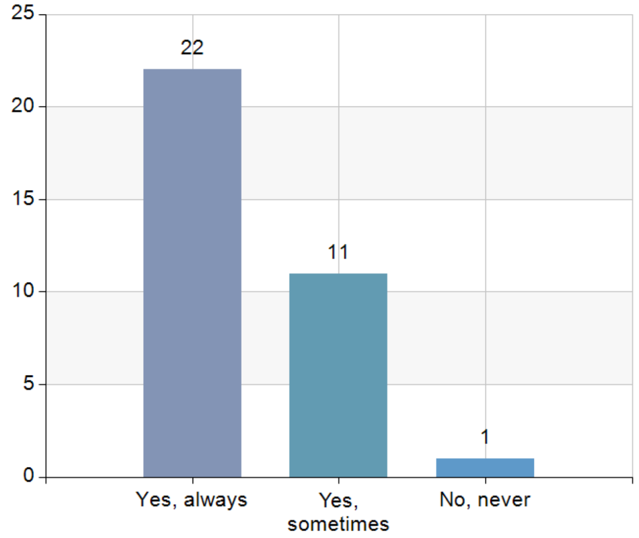


• Is intraoperative spinal navigation for spinal injuries in use at your department?

| Namn | Antal | % |
|---|---|---|
| Yes, always | 11 | 31,4 |
| Yes, sometimes | 21 | 60 |
| No, never | 3 | 8,6 |
| **Total** | **35** | **100** |

| Svarsfrekvens |
|---|
| 100% (35/35) |

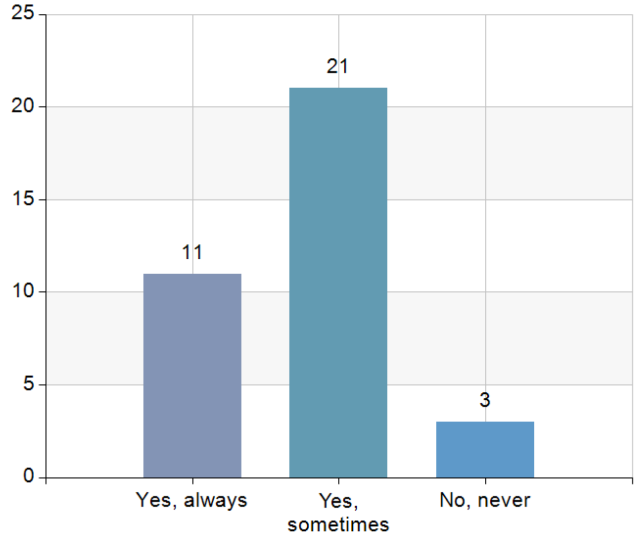


• Is lumbar drain applied to lower the intraspinal pressure in patients with SCI?

| Namn | Antal | % |
|---|---|---|
| Yes, always | 1 | 2,9 |
| Yes, sometimes | 3 | 8,6 |
| No, never | 31 | 88,6 |
| **Total** | **35** | **100** |

| Svarsfrekvens |
|---|
| 100% (35/35) |

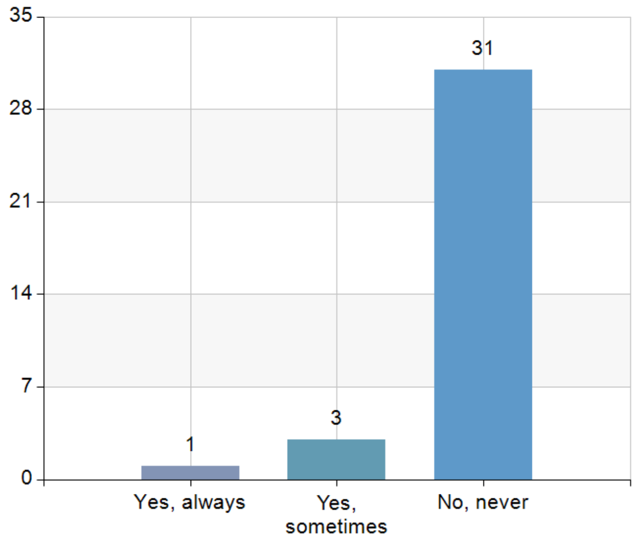


• If so, is a specific spinal perfusion pressure targeted?

| Namn | Antal | % |
|---|---|---|
| Yes, always | 0 | 0 |
| Yes, sometimes | 0 | 0 |
| No, never | 1 | 100 |
| **Total** | **1** | **100** |

| Svarsfrekvens |
|---|
| 2,9% (1/35) |

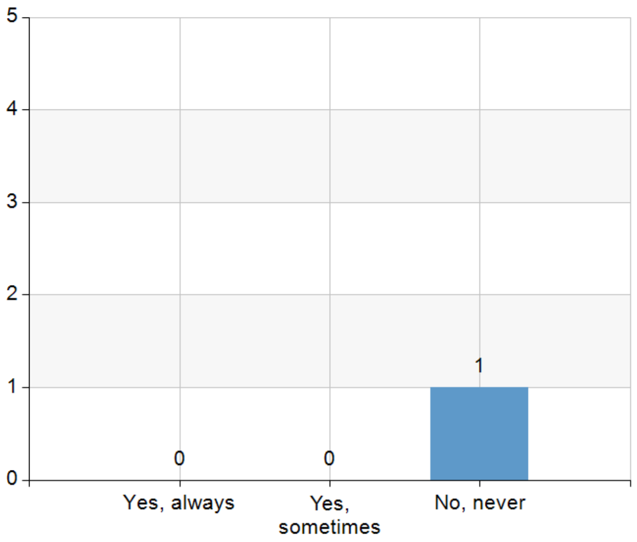


• Is durotomy performed at your institution to lower the intraspinal pressure?

| Namn | Antal | % |
|---|---|---|
| Yes, always | 0 | 0 |
| Yes, sometimes | 1 | 2,9 |
| No, never | 34 | 97,1 |
| **Total** | **35** | **100** |

| Svarsfrekvens |
|---|
| 100% (35/35) |

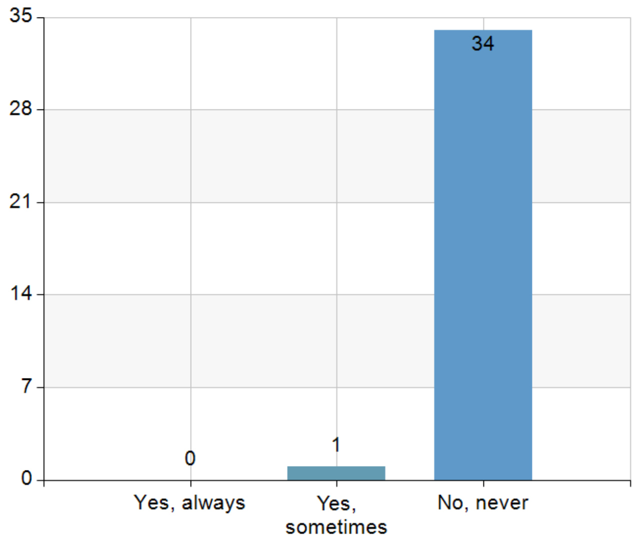

• Do you have any comments related to the questions?

| |
|---|
| We tend to start with duroplastic soon. |
| I can’t answer the questions regarding the cervical spine because evaluation and treatment of these injuries is performed by the neurosurgeons |
| Timing of surgery based on the progression of neurologic deficits rather than the severity. |

• Does your hospital have a management protocol for thromboprophylactic medication in patients with SCI?

| Namn | Antal | % |
|---|---|---|
| Yes | 34 | 97,1 |
| No | 1 | 2,9 |
| **Total** | **35** | **100** |

| Svarsfrekvens |
|---|
| 100% (35/35) |

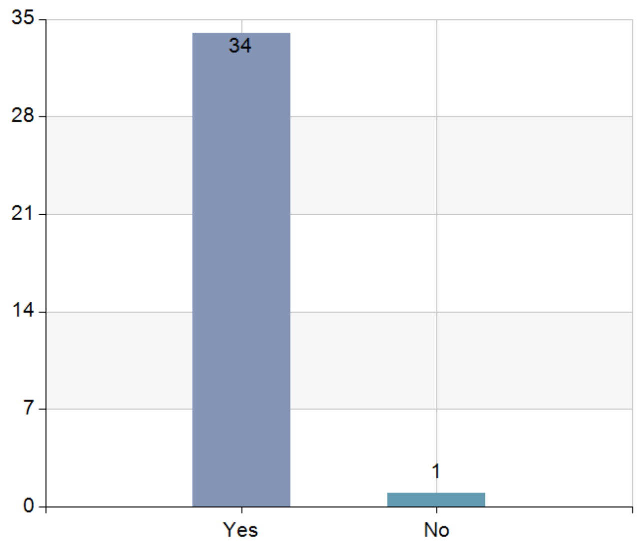


• What is your department's drug of choice for thromboprofylaxis in hospitalized patients with SCI?

| Namn | Antal | % |
|---|---|---|
| Low-molecular-weight heparin | 33 | 97,1 |
| Heparin infusion | 0 | 0 |
| Direct oral anticoagulants | 0 | 0 |
| Warfarin | 0 | 0 |
| Other - Please specify | 1 | 2,9 |
| **Total** | **34** | **100** |

| Svarsfrekvens |
|---|
| 97,1% (34/35) |

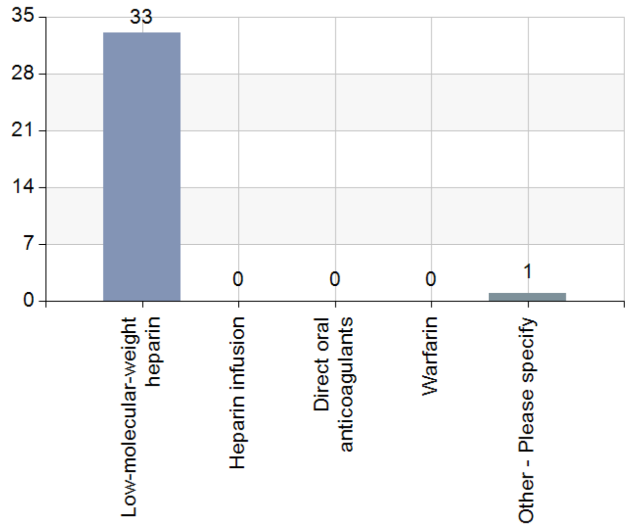


| **Other - Please specify** |
|---|
| SCD |

• Given that a patient has only a SCI, at what time point from completed surgery do you consider it safe to start thromboprophylactic treatment?

| Namn | Antal | % |
|---|---|---|
| <24 hours | 17 | 50 |
| 24-48 hours | 15 | 44,1 |
| 48-72 hours | 2 | 5,9 |
| >72 hours | 0 | 0 |
| **Total** | **34** | **100** |

| Svarsfrekvens |
|---|
| 97,1% (34/35) |

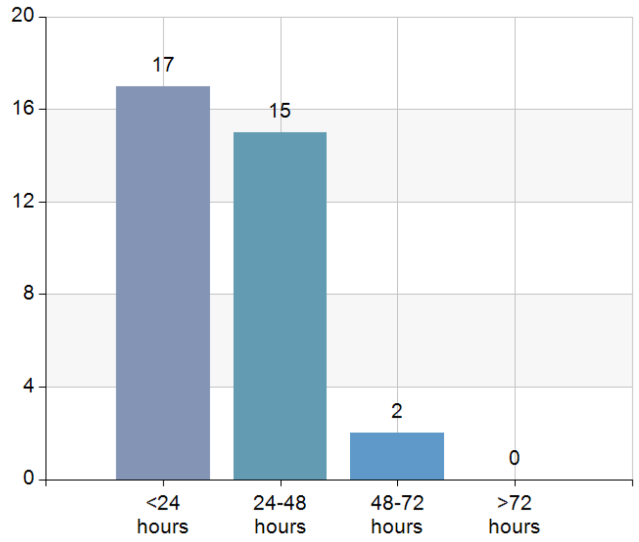


- Does your department apply stockings on a routine basis for patients with SCI and neurological deficits?

| Namn | Antal | % |
|---|---|---|
| Yes, always | 18 | 51,4 |
| Yes, sometimes | 12 | 34,3 |
| No, never | 5 | 14,3 |
| **Total** | **35** | **100** |

| Svarsfrekvens |
|---|
| 100% (35/35) |

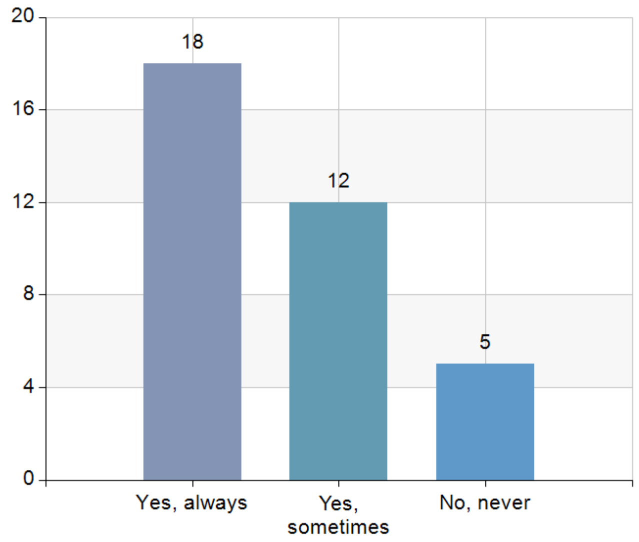


- Do you have any comments related to the questions?

| |
|---|
| Intermittent pneumatic leg compression (intermittent vadkompression, ”benpump”) vår vanligaste metod för trombosprofylax under operation i nacke och rygg, även vid elektiva operationer. |

- What is the routine radiological investigation for the initial post-operative control?

| Namn | Antal | % |
|---|---|---|
| X-Ray | 11 | 31,4 |
| CT | 24 | 68,6 |
| MRI | 0 | 0 |
| **Total** | **35** | **100** |

| Svarsfrekvens |
|---|
| 100% (35/35) |

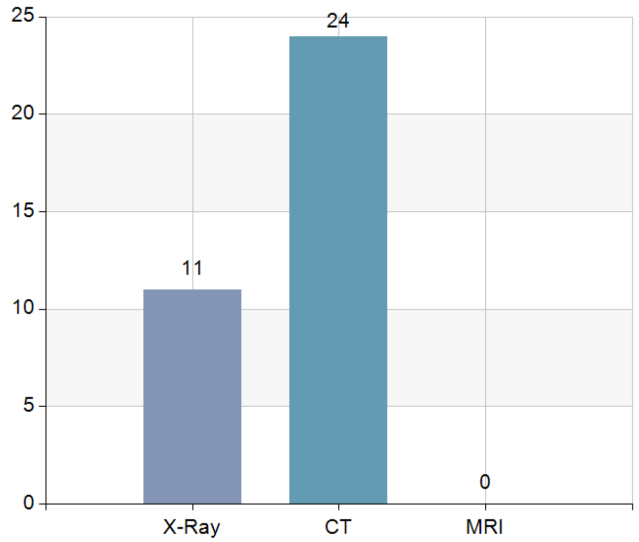


• In operated patients with SCI and C-spine fractures: Are cervical collars routinely applied post-operatively?

| Namn | Antal | % |
|---|---|---|
| Yes, always | 6 | 17,1 |
| Yes, sometimes | 17 | 48,6 |
| No, never | 8 | 22,9 |
| Not applicable | 4 | 11,4 |
| **Total** | **35** | **100** |

| Svarsfrekvens |
|---|
| 100% (35/35) |

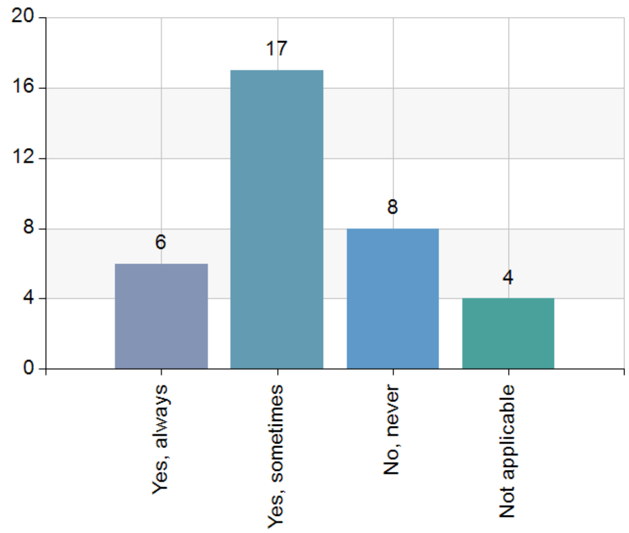


• If so, for how long, on average, are collars applied?

| Namn | Antal | % |
|---|---|---|
| 2 weeks | 0 | 0 |
| 2-4 weeks | 3 | 13 |
| 4-6 weeks | 3 | 13 |
| 6-8 weeks | 9 | 39,1 |
| 8-12 weeks | 7 | 30,4 |
| >12 weeks | 1 | 4,3 |
| **Total** | **23** | **100** |

| Svarsfrekvens |
|---|
| 65,7% (23/35) |

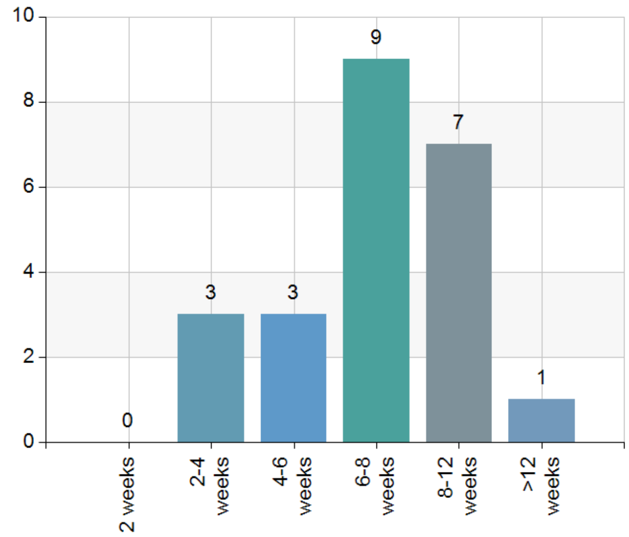


• Are operated patients with SCI undergoing regular follow-up controls in your department?

| Namn | Antal | % |
|---|---|---|
| Yes, always | 26 | 74,3 |
| Yes, sometimes | 9 | 25,7 |
| No, never | 0 | 0 |
| **Total** | **35** | **100** |

| Svarsfrekvens |
|---|
| 100% (35/35) |

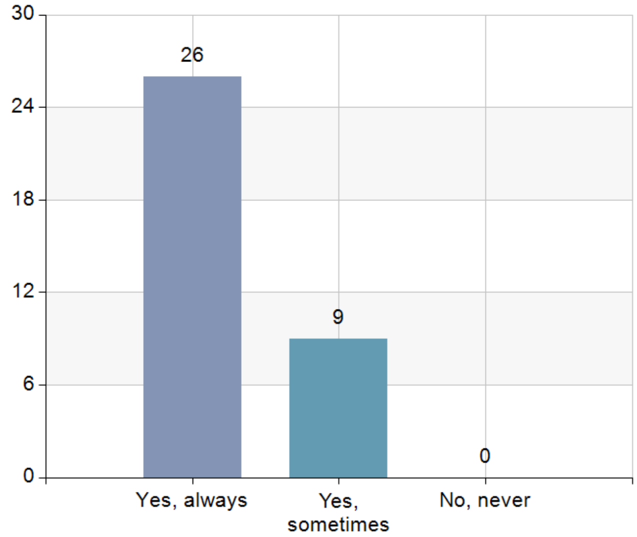


• At discharge, is the neurological status of the patient recorded and documented?

| Namn | Antal | % |
|---|---|---|
| Yes, always | 24 | 68,6 |
| Yes, sometimes | 11 | 31,4 |
| No, never | 0 | 0 |
| **Total** | **35** | **100** |

| Svarsfrekvens |
|---|
| 100% (35/35) |

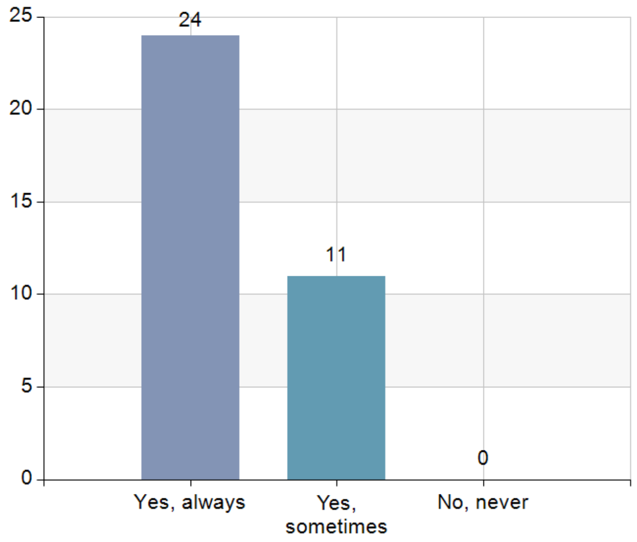


• Do you have any comments related to the questions?

| |
|---|
| Continued FU is mainly at the Dept Rehab med, but at least one FU including radiological examination at our dept. The neurological status at discharge is evaluated of a specialized team from the Dept Rehabilitionmedcine (physioterapeut + physician) |
| We have some slip, but all pt SCI should have complete AISA scoore before discharge |

• Are elevated levels of MAP considered equally important for patients with CCS compared to "regular" SCI in your department?

| Namn | Antal | % |
|---|---|---|
| Yes, always | 7 | 20,6 |
| Yes, sometimes | 15 | 44,1 |
| No, never | 6 | 17,6 |
| Not applicable | 6 | 17,6 |
| **Total** | **34** | **100** |

| Svarsfrekvens |
|---|
| 97,1% (34/35) |

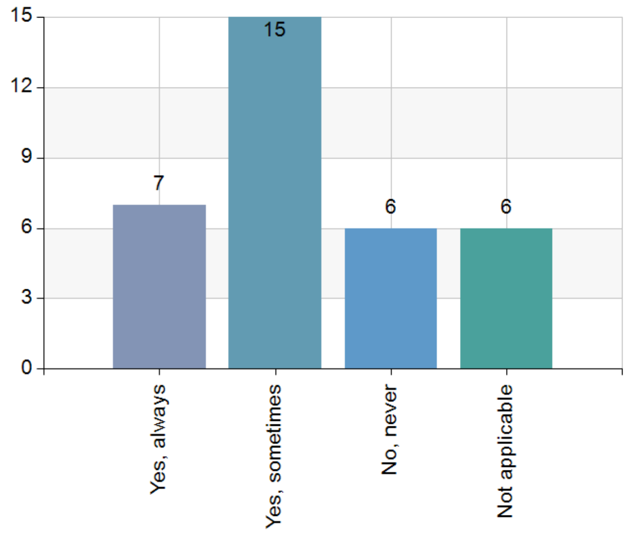


• If so, are patients with CCS admitted to ICU for observation and inotropic medications?

| Namn | Antal | % |
|---|---|---|
| Yes, always | 9 | 40,9 |
| Yes, sometimes | 12 | 54,5 |
| No, never | 1 | 4,5 |
| **Total** | **22** | **100** |

| Svarsfrekvens |
|---|
| 62,9% (22/35) |

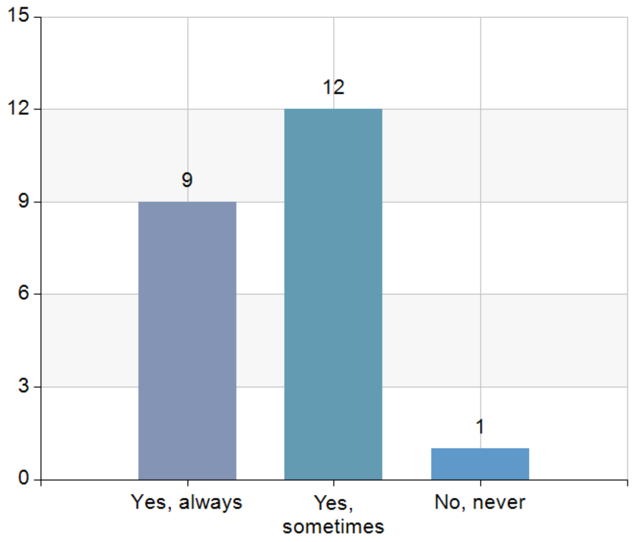


• Is timing of surgery for CCS considered along the same guidelines as SCI patients in your department?

| Namn | Antal | % |
|---|---|---|
| Yes, always | 10 | 29,4 |
| Yes, sometimes | 19 | 55,9 |
| No, never | 0 | 0 |
| Not applicable | 5 | 14,7 |
| **Total** | **34** | **100** |

| Svarsfrekvens |
|---|
| 97,1% (34/35) |

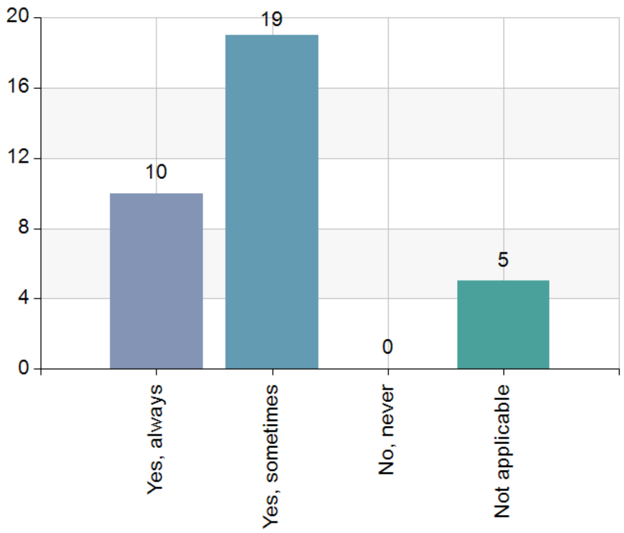


• Do you have any comments related to the questions?

| |
|---|
| I can’t answer the questions regarding the cervical spine because evaluation and treatment of these injuries is performed by the neurosurgeons |
| Not involved |
| Dept policy: CCS should be considered for decompression <48 hrs after trauma |
| CCS usually treated by neurosurgeons and SCI by orthopedic surgeons. |

• If you have finished answering the questions, please select "SUBMIT" below and then continue to the next page to submit your answers. Please note that this will lock and submit the survey and no further changes will be possible. • If you need more time to take the survey, you can continue at a later time. To do so, you can click the pause button at the bottom of the page. You can then resume taking the survey by following the link in the invitation e-mail that was sent to you earlier.

| Namn | Antal | % |
|---|---|---|
| SUBMIT | 24 | 100 |
| **Total** | **24** | **100** |

| Svarsfrekvens |
|---|
| 100% (24/24) |

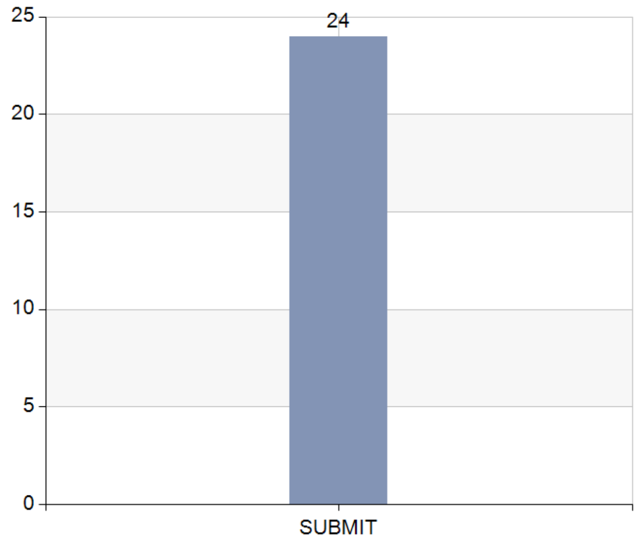


• On average, what is the number of days from injury until transfer to rehabilitation?

| Namn | Antal | % |
|---|---|---|
| <1 week | 2 | 12,5 |
| 1-2 weeks | 7 | 43,8 |
| >2 weeks | 7 | 43,8 |
| **Total** | **16** | **100** |

| Svarsfrekvens |
|---|
| 100% (16/16) |

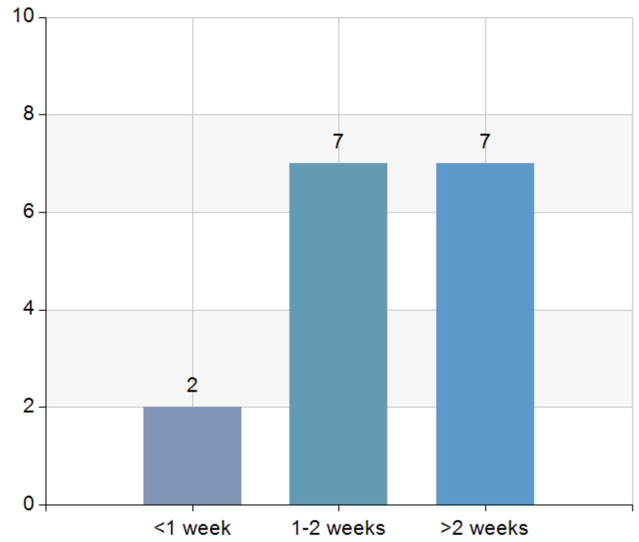


• Does rehabilitation take place in a spinal unit with only or predominantly spinal cord injuries?

| Namn | Antal | % |
|---|---|---|
| Yes, always | 11 | 68,8 |
| Yes, sometimes | 3 | 18,8 |
| No, never | 2 | 12,5 |
| **Total** | **16** | **100** |

| Svarsfrekvens |
|---|
| 100% (16/16) |

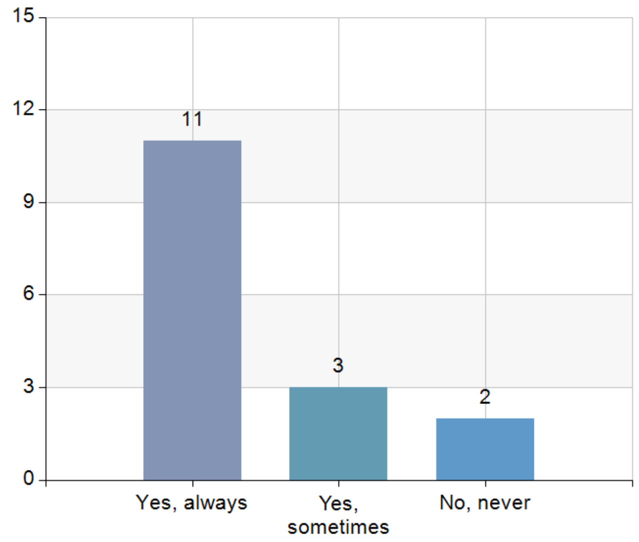

• Which medical specialty is in charge of the rehabilitation?

| Namn | Antal | % |
|---|---|---|
| Neurologist | 5 | 31,2 |
| Physical medicine and rehabilitation physician | 11 | 68,8 |
| Other - Please specify | 0 | 0 |
| **Total** | **16** | **100** |

| Svarsfrekvens |
|---|
| 100% (16/16) |

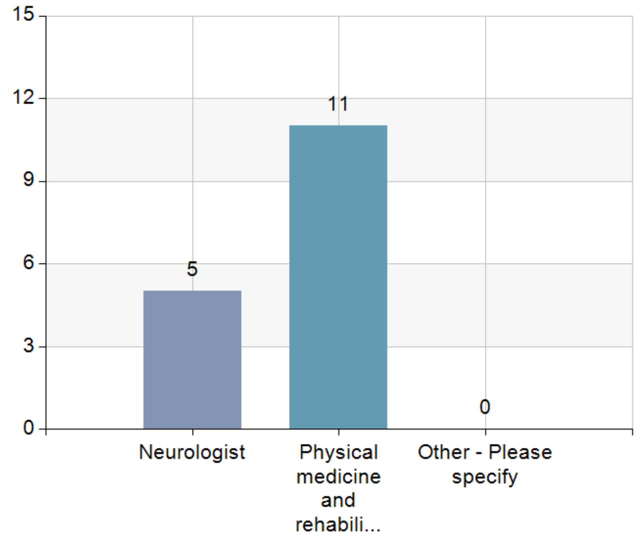


• Are there one or more advanced physical devices that improves mobilization such as therapy pool, body weight supported treadmill, or exoskeleton available at your institution?

| Namn | Antal | % |
|---|---|---|
| Yes | 12 | 75 |
| No | 4 | 25 |
| **Total** | **16** | **100** |

| Svarsfrekvens |
|---|
| 100% (16/16) |

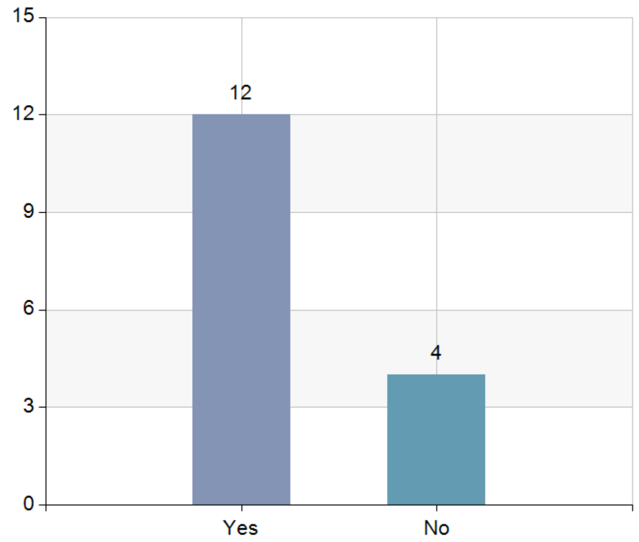


• Is your unit or program accredited according to international standards like ISO or CARF?

| Namn | Antal | % |
|---|---|---|
| Yes | 7 | 46,7 |
| No | 8 | 53,3 |
| **Total** | **15** | **100** |

| Svarsfrekvens |
|---|
| 93,8% (15/16) |

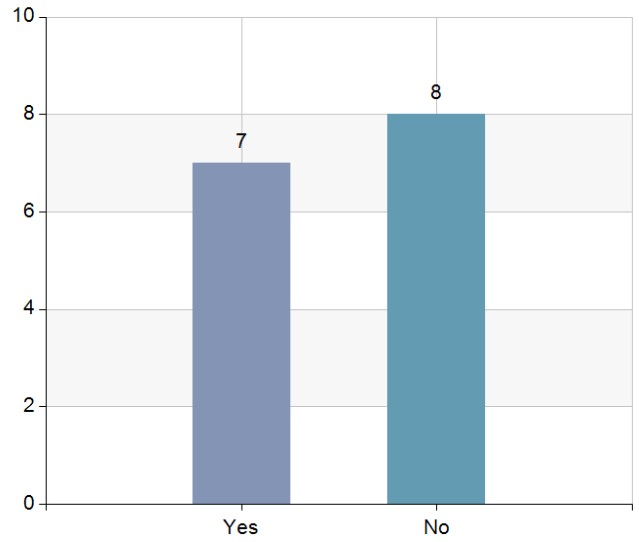


• Is systematic registration of data based on international data sets implemented in your unit or program (e.g., NordicSCIR)?

| Namn | Antal | % |
|---|---|---|
| Yes, always | 15 | 93,8 |
| Yes, sometimes | 0 | 0 |
| No, never | 1 | 6,2 |
| **Total** | **16** | **100** |

| Svarsfrekvens |
|---|
| 100% (16/16) |

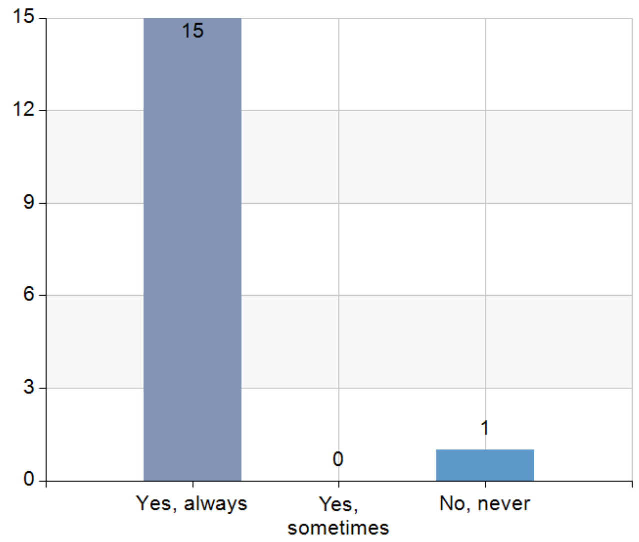


• Do you have any comments related to the questions?

| |
|---|
| Question no 1 concerning timing is difficult to answer. Depends on the kind, level and consequences of injury. Cervical injuries with respiratory impairment and need for intensive care as well as other aptients with need of intensive care might take longer time before transfer to our unit is possible. ”Uncomplicated” patients can often be transferred within one week. |
| In Region Örebro län we send all our tSCI-patients to Uppsala for primary rehabilitation and they go there straight from the acute orthopedic ward, without passing our department. So the first two questions above therefor applies to Uppsala. But the following questions apply to our ward. |
| Apart from PM&R specialists there are sometimes neurosurgeons or neurologists who might be in charge of the acute SCI ward.<br><br>In one of our rehabilitation units the SCI patients are in the same ward as other neurorehabilitaion patients (stroke, traumatic braininjury etc) |

Question 2: We do not have a specialized Spinal Unit in Iceland (the population of the country, i.e., the catchment area, is by far too small to allow that. Instead, we have a specialized SCI team within the department of rehabilitation medicine

Last question: The registration of data based on international data sets (last question) is mainly due to our participatoin in NordicSCIR (The Nordic Spinal Cord Injury Registry).
Otherwise, we have "traditional" medical records .
4th question (the third in chapter 2): But we have a warm large therapy pool, Lokomat computer-assisted treadmill for locomotion training, and a well equipped physiotherapy facility.

• In patients with SCI with permanent ASIA A, B, or C, for how long is routinely thromboprophylactic treatment considered necessary?

| Namn | Antal | % |
|---|---|---|
| <1 month | 0 | 0 |
| 1-3 months | 14 | 87,5 |
| 4-6 months | 2 | 12,5 |
| 7-12 months | 0 | 0 |
| Life-long | 0 | 0 |
| **Total** | **16** | **100** |

| Svarsfrekvens |
|---|
| 100% (16/16) |

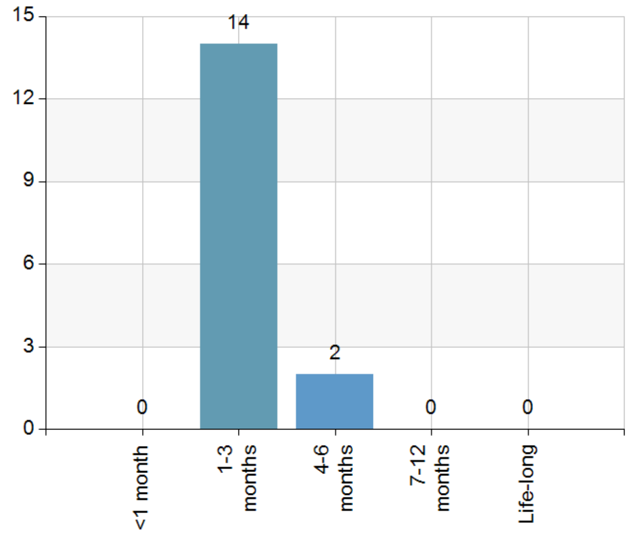


• In your institution, what is the drug of choice for long term (>2 months) treatment?

| Namn | Antal | % |
|---|---|---|
| Low-molecular-weight heparin | 15 | 93,8 |
| Direct oral antithrombotics | 1 | 6,2 |
| Warfarin | 0 | 0 |
| Other - Please specify | 0 | 0 |
| **Total** | **16** | **100** |

| Svarsfrekvens |
|---|
| 100% (16/16) |

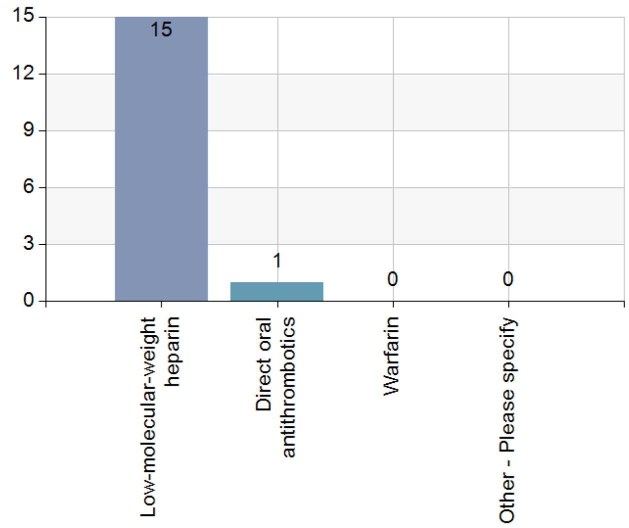


• Do you have any comments related to the questions?

| |
|---|
| We change to direct oral antithrombotics if the patients gets a complication with pulmonary embolous, deep vein thrombosis. |
| When OTHER REASONS than SCI necessitate longer treatment duration than 12 weeks / 3 months, (such as e.g. repeated pulmonary embolism), then peroral anticoagulatoin is started (usually direct oral antithrombotics, more seldom Warfarin). But this is the exception - the main rule is that 3 months of anticoagulation (with Fragmin low molecular Heparin) is appropriate, so that most patients continue with Fragmin for the third month as well. In mild injuries who quickly regain near full mobility, we can have shorter duration of thromboprophylactic treatment. |

- Are patients with SCI (Spinal Cord Injury) followed up at regular intervals in your department?

| Namn | Antal | % |
|---|---|---|
| Yes, always | 16 | 100 |
| Yes, sometimes | 0 | 0 |
| No, never | 0 | 0 |
| **Total** | **16** | **100** |

| Svarsfrekvens |
|---|
| 100% (16/16) |

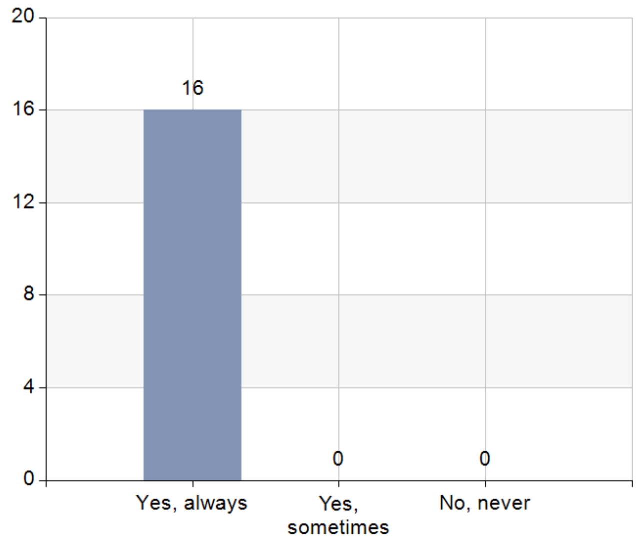


- If so, for how long does routine follow-up occur after initial injury?

| Namn | Antal | % |
|---|---|---|
| <1 year | 3 | 18,8 |
| 1-2 years | 4 | 25 |
| 2-5 years | 1 | 6,2 |
| 5-10 years | 0 | 0 |
| Life-long | 8 | 50 |
| **Total** | **16** | **100** |

| Svarsfrekvens |
|---|
| 100% (16/16) |

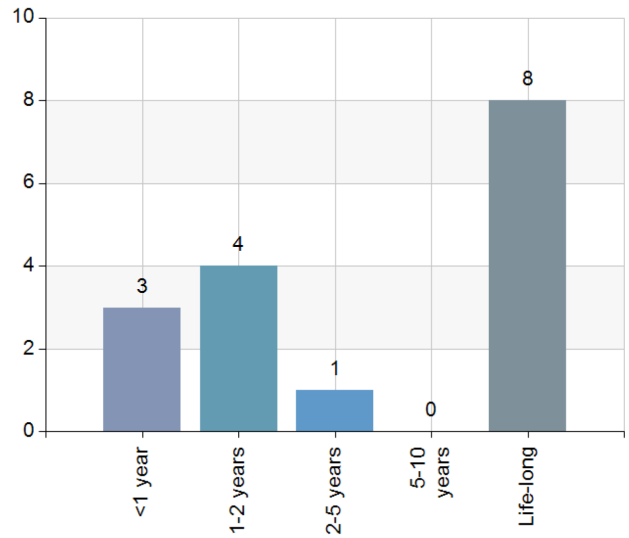


- Do you have any comments related to the questions?

| |
|---|
| We do our follow-ups are every third year. |

Lifelong follow-up, first annually, then with gradually increasing interval with up to 5 years between visits if everything goes well, mainly for the more severe cases, that have a neurogenic bladder paralysis, and need to go through neuro-urologic exam (cystometry, sometimes also cystoscopy, or only cystoscopy for those with indwelling suprapubic catheter) and ultrasound examination of kidneys, and these patients also meet all team mmebers (doctor, nures, physioth,ergother., social worker, psychologist if needed) during a 1 1/2 days visit to the daycare ward.
The patients who have retained (or regain) their volontary voiding ability, and also usually have much less severe SCI, are referred to primary care, and they are also offered to take contact and meet their doctor at the outpatient ward as needed (but unfortunately only on an a-needed bases, not on a regular basis for all patients, mainly due to lack of doctor's manpower at our clinic).

• Do you have access to treatment with botulinum toxin injections in your unit/hospital?

| Namn | Antal | % |
|---|---|---|
| Yes, always | 16 | 100 |
| Yes, sometimes | 0 | 0 |
| No, never | 0 | 0 |
| **Total** | **16** | **100** |

| Svarsfrekvens |
|---|
| 100% (16/16) |

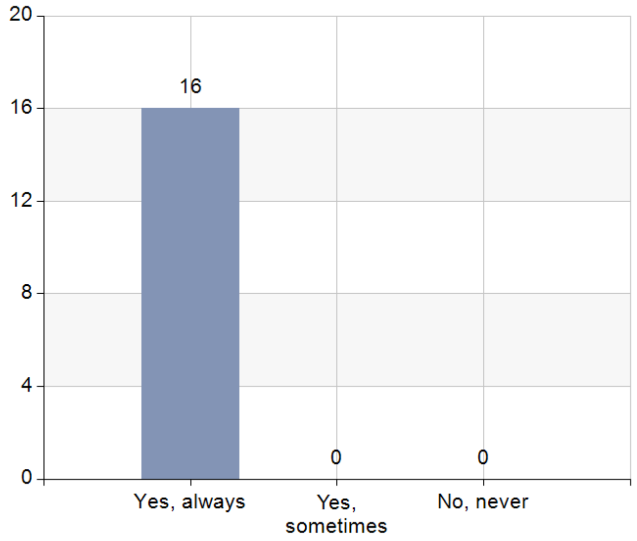


• Do you have access to Baclofen pump refilling and adjustment in your unit/hospital?

| Namn | Antal | % |
|---|---|---|
| Yes, always | 15 | 93,8 |
| Yes, sometimes | 1 | 6,2 |
| No, never | 0 | 0 |
| **Total** | **16** | **100** |

| Svarsfrekvens |
|---|
| 100% (16/16) |

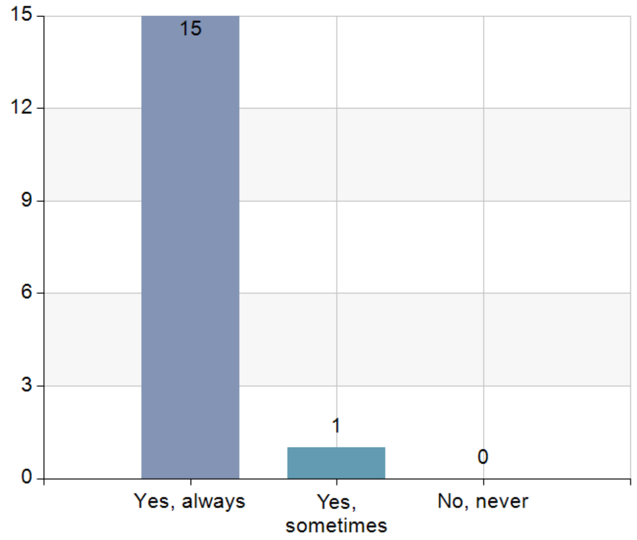


• Do you have any comments related to the questions?

• Do you have a pediatric unit with special expertise in children with spinal cord injuries?

| Namn | Antal | % |
|---|---|---|
| Yes, always | 6 | 37,5 |
| Yes, sometimes | 3 | 18,8 |
| No, never | 7 | 43,8 |
| **Total** | **16** | **100** |

| Svarsfrekvens |
|---|
| 100% (16/16) |

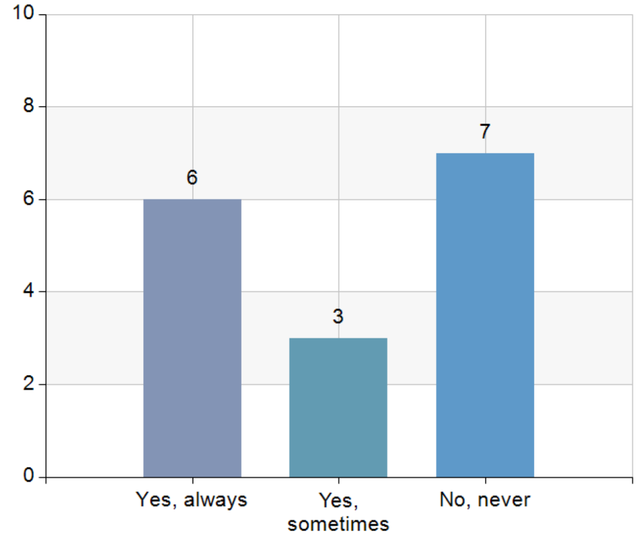


• Do you have any comments related to the question?

| |
|---|
| Young adults transfer to regional SCI rehabilitation center. Younger children remain at pediatric hospital with SCI rehabilitation expertise on consultational basis. |
| The youngest childern (up to aprox. 5-7 years are taken care of atthe Pediatric clinic.<br>The older children (<5-7 years approx. ) usually start at the pediatric ward during the acute phase, then they are transferred to a small pediatric rehab unit, but come to the day care ward of the rehabilitation clinic (where specialized SCI rehabilitation is done) for the specilized SCI training (PT, OT etc.) for most part of their SCI rehabilitation. Then, they get specialized training and advice, but do not need to sleep and stay around the clock among adult and sometimes quite old other patients. |

• Do you have access to urodynamic examinations in your unit/hospital?

| Namn | Antal | % |
|---|---|---|
| Yes, always | 15 | 93,8 |
| Yes, sometimes | 1 | 6,2 |
| No, never | 0 | 0 |
| **Total** | **16** | **100** |

| Svarsfrekvens |
|---|
| 100% (16/16) |

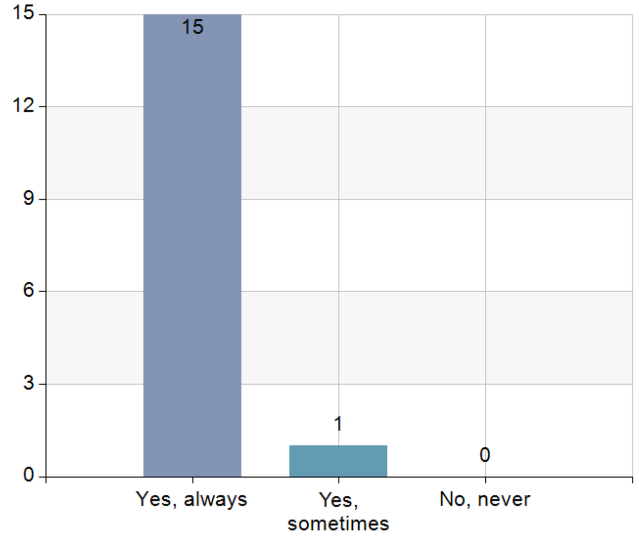


• What is the approximate percentage of patients discharged with indwelling catheter (urethral or suprapubic)?

| Namn | Antal | % |
|---|---|---|
| <25% | 13 | 81,2 |
| 25-30% | 2 | 12,5 |
| 50-75% | 1 | 6,2 |
| >75% | 0 | 0 |
| **Total** | **16** | **100** |

| Svarsfrekvens |
|---|
| 100% (16/16) |

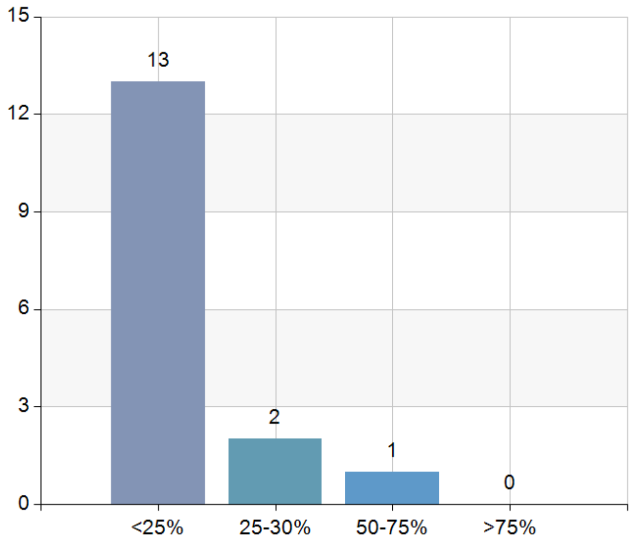


• At your department, do you have specialized personnel for:

- Urinary problems (i.e., urotherapists)?

| Namn | Antal | % |
|---|---|---|
| Yes | 14 | 87,5 |
| No | 2 | 12,5 |
| **Total** | **16** | **100** |

| Svarsfrekvens |
|---|
| 100% (16/16) |

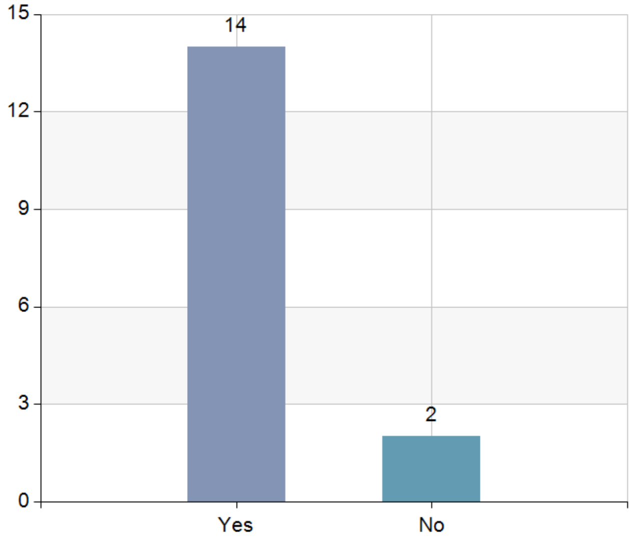


- Sexual counseling?

| Namn | Antal | % |
|---|---|---|
| Yes | 16 | 100 |
| No | 0 | 0 |
| **Total** | **16** | **100** |

| Svarsfrekvens |
|---|
| 100% (16/16) |

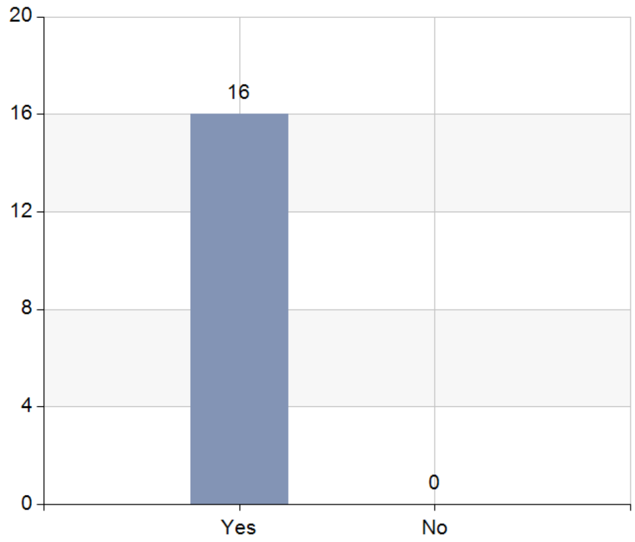


- Bowel management?

| Namn | Antal | % |
|---|---|---|
| Yes | 14 | 87,5 |
| No | 2 | 12,5 |
| **Total** | **16** | **100** |

| Svarsfrekvens |
|---|
| 100% (16/16) |

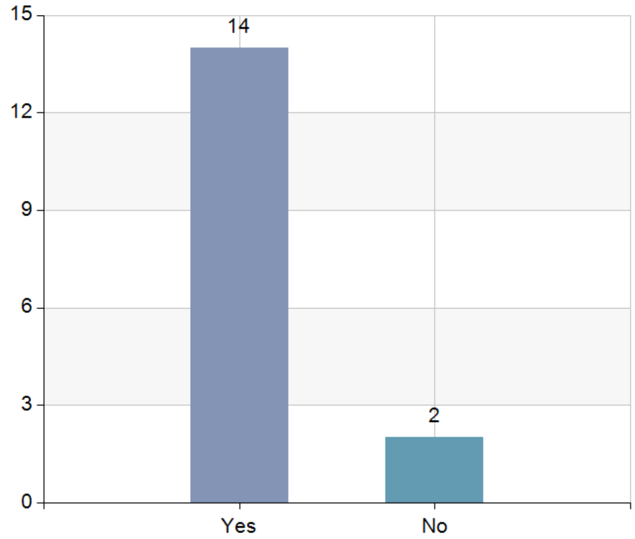


• Do you have any comments related to the questions?

| |
|---|
| One of our nurses has extended knowledge of bladder and bowel management after tSCI. |
| Only 0-5 patients annually are discharged with indwelling catheter. |

The last question: I am not sure how to answer this question. We have considerable expertise in all three areas of concern among the team members or the ward, which in almost all cases is sufficient to solve most, if not all, or problems.
We also have very good and direct ACCESS to even more specialized staff which are dedicated to these issues (but not always with a focus of the specialized problems related to SCI!). However, these persons are NOT located in our small department of rehabilitation medicine, they work in other departments of the hospital (e.g., urotherapists at the neuro-dynamic lab., where cystometries, cystoscopies, suprapubic catheter insertions etc. are performed; stoma therapeuts that work at the gastroenterology unit within the department of internal medicine, that are concerned with bowel management, mainly but not only in connection with colostomies etc.,; as well as possibility to councel a sexolog, which works as a consultant for the whole hospital, and covers sexual issues in disease in general, not only the specific problems that occur in patients with SCI).
So, we are a little ”in between” - however, I answered yes, as I and my co-workers (not the least the specialized nurse at the day care department who organizes and coordinates all activities in connection with the regular SCI follow ups mentioned above, as well as the most experienced nurses in the inpatient ward, who take care of most of the primary SCI rehabilitation period) can in almost all cases, function as ”specialized personnel” that can give adequate advice to our patients.

• What is the percentage of patients discharged directly home?

| Namn | Antal | % |
|---|---|---|
| <25% | 2 | 12,5 |
| 25-50% | 2 | 12,5 |
| 50-75% | 8 | 50 |
| >75% | 4 | 25 |
| **Total** | **16** | **100** |

| Svarsfrekvens |
|---|
| 100% (16/16) |

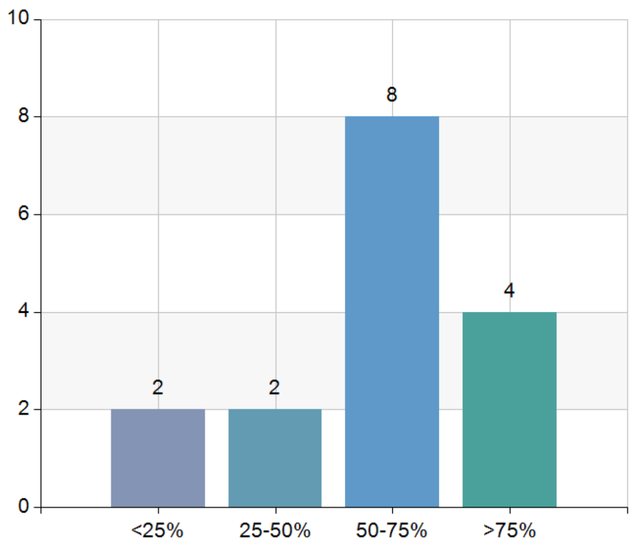


• Following SCI ASIA A-D, what is the average length of stay in your department?

| Namn | Antal | % |
|---|---|---|
| <1 month | 1 | 6,2 |
| 1-3 months | 12 | 75 |
| 4-6 months | 3 | 18,8 |
| **Total** | **16** | **100** |

| Svarsfrekvens |
|---|
| 100% (16/16) |

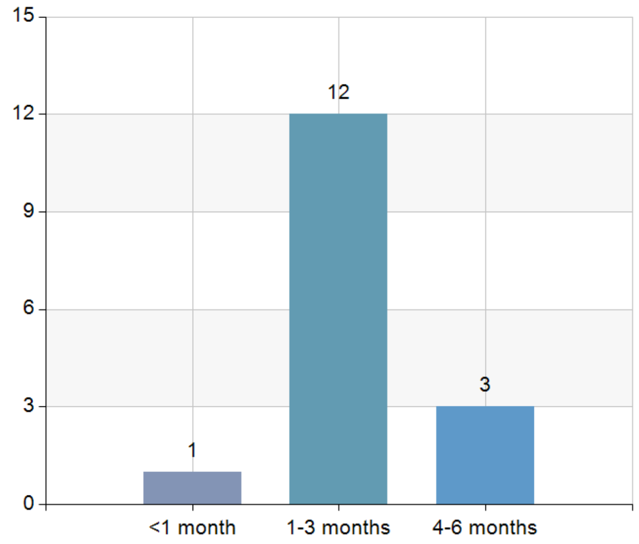


• Do you have any comments related to the questions?

| |
|---|
| The last question: Very much dependent on the level of injury. A low complete injury (ASIA A) might need shorter hospitalisation. |
| At our ward we give patients secondary rehabilitation after transfer from Uppsala (where they treat patients with tetraplegia 10-12 weeks and paraplegia 6-8 weeks). Our LOS is a few weeks shorter, respectively. |
| First question: Percentage discharged home varies: often >75%, but unfortunately, when we looked back the last 4-5 years, more often slightly more than 25% needed to go either to other wards (mainly malignant tumor metastases to the spinal cord, that go back to the oncologic ward, or sometimes to terminal care), or directly to a nursing home (as personal assistance (Sw. Personlig assistans), and home nursing, are unfortunately less developed in Iceland than in the other Scandinavian countries).. <br><br> 4-6 months OR MORE ... cervical injuries probably have 6-9 months average length of stay (and the high or skyhigh tetraplegcs sometimes need considerably longer durations of stay - up to a year or more!). |

• If you have finished answering the questions, please select "SUBMIT" below and then continue to the next page to submit your answers. Please note that this will lock and submit the survey and no further changes will be possible. • If you need more time to take the survey, you can continue at a later time. To do so, you can click the pause button at the bottom of the page. You can then resume taking the survey by following the link in the invitation e-mail that was sent to you earlier.

| Namn | Antal | % |
|---|---|---|
| SUBMIT | 12 | 100 |
| **Total** | **12** | **100** |

| Svarsfrekvens |
|---|
| 100% (12/12) |

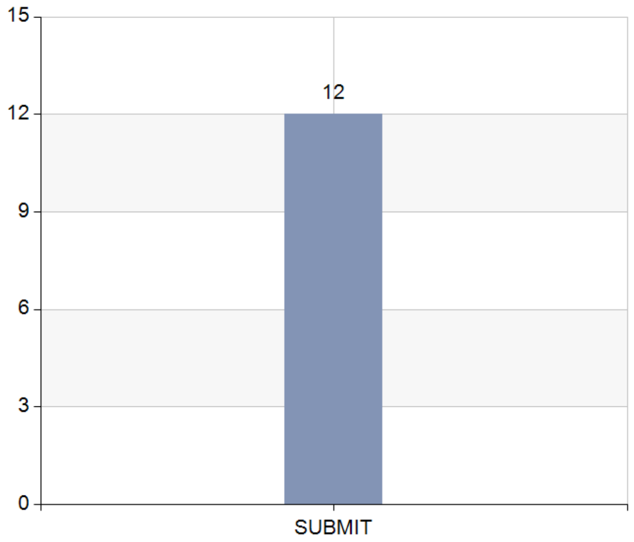

Supplement: Supplementary file 1 — Supplementary Material 1. [file 13049_2025_1349_MOESM1_ESM.pdf]
